# The *Drosophila* hnRNP F/H homolog Glorund recruits dFMRP to inhibit *nanos* translation elongation

**DOI:** 10.1093/nar/gkac500

**Published:** 2022-06-14

**Authors:** Yingshi Peng, Elizabeth R Gavis

**Affiliations:** Department of Molecular Biology, Princeton University, Princeton, NJ 08544, USA; Department of Molecular Biology, Princeton University, Princeton, NJ 08544, USA

## Abstract

Translational control of maternal mRNAs generates spatial and temporal patterns of protein expression necessary to begin animal development. Translational repression of unlocalized *nanos* (*nos*) mRNA in late-stage *Drosophila* oocytes by the hnRNP F/H homolog, Glorund (Glo), is important for embryonic body patterning. While previous work has suggested that repression occurs at both the translation initiation and elongation phases, the molecular mechanism by which Glo regulates *nos* translation remains elusive. Here, we have identified the *Drosophila* fragile X mental retardation protein, dFMRP, as a Glo interaction partner with links to the translational machinery. Using an oocyte-based *in vitro* translation system, we confirmed that Glo regulates both initiation and elongation of a *nos* translational reporter and showed that dFMRP specifically represses translation elongation and promotes ribosome stalling. Furthermore, we combined mutational analysis and *in vivo* and *in vitro* binding assays to show that Glo's qRRM2 domain specifically and directly interacts with dFMRP. Our findings suggest that Glo regulates *nos* translation elongation by recruiting dFMRP and that Glo's RNA-binding domains can also function as protein-protein interaction interfaces critical for its regulatory functions. Additionally, they reveal a mechanism for targeting dFMRP to specific transcripts.

## INTRODUCTION

Translational control, coupled with other types of post-transcriptional regulation including mRNA localization and degradation, is a key mechanism for establishing and maintaining protein asymmetries. Translational control plays a particularly important role in early embryonic development in organisms like *Drosophila* (1,2), *Caenorhabditis elegans* (3) and *Xenopus* (1), where transcription of the zygotic genome is delayed for many cell divisions. The embryo therefore relies on spatial and temporal control over the translation of a stockpile of maternally synthesized mRNAs to execute the earliest cell fate determination and axial patterning programs.

The *Drosophila* anterior-posterior body axis is patterned by opposing protein gradients of Bicoid (Bcd) and Nanos (Nos) in the early embryo. Both gradients originate from localized translation of maternal *bcd* and *nos* mRNAs at the anterior and posterior poles of the embryo, respectively (4). Unlike *bcd*, which is tightly sequestered at the anterior pole (5), only a small proportion (4%) of *nos* resides at the posterior and is translated (6). Therefore, whereas RNA localization is sufficient to sequester Bcd protein synthesis at the anterior (7), an additional level of control is required to restrict production of Nos to the posterior. This is accomplished by maintaining *nos* in a translationally repressed state that is alleviated only by its posterior localization (6,8,9). Failure to repress unlocalized *nos* mRNA and the consequent accumulation of Nos throughout the embryo result in loss of anterior structures (10,11).

As a maternally supplied mRNA, *nos* is synthesized in the ovarian nurse cells (12) and then transferred to the oocyte during the latter part of oogenesis, where localization ensues. *nos* is translated initially in the nurse cells but becomes repressed when it enters the oocyte. Upon localization to the posterior of the oocyte, translation is reactivated (9). Translational repression of the unlocalized *nos* is mediated primarily by a 90-nucleotide *cis*-acting translational control element (TCE) in the *nos* 3′ untranslated region (3′ UTR) (13–15). The *nos* TCE consists of a base stem, TCEI, connected to two stem loops, termed TCEII and TCEIII, which have temporally distinct activities (9,16). TCEI and TCEIII together confer translational regulation on *nos* during late oogenesis through their interaction with the hnRNP F/H protein Glorund (Glo) (16–18). TCEII confers repression on unlocalized *nos* during early embryogenesis through its interaction with a different RNA-binding protein, Smaug (Smg) (15,19).

Translational repression of unlocalized *nos* during late stages of oogenesis appears to be bipartite. Previous *in vitro* translation experiments showed that TCE-mediated repression of a reporter RNA in embryo extract was abolished if the reporter RNA was unable to bind eukaryotic translation initiation factor 4E (eIF4E) (20). This cap-dependence of repression suggests that the TCE acts to block initiation in the embryo and is consistent with the ability of Smg to recruit the *Drosophila* 4E-binding protein (4E-BP), Cup, and disrupt the formation of the translation initiation complex eIF4F (21). By contrast, TCE-mediated repression of the same reporter in ovary extract was only partially abrogated (20), indicating that repression in the ovary is only cap-dependent in part. Furthermore, sucrose density gradient sedimentation showed that approximately 50% of *nos* is polysome-associated whereas only 4% of *nos* is posteriorly localized and translationally active (22), consistent with repression at the elongation step. Compared to initiation control, an elongation-based mechanism could afford a more rapid on/off switch to shut down translation as *nos* enters the oocyte and then reactivate translation as it becomes localized.

Glo is the only known repressor of *nos* in the ovary, suggesting that it might mediate both the initiation and elongation-based repression mechanisms. Like its mammalian counterparts, Glo is a multi-functional RNA-binding protein. In addition to translationally repressing *nos*, Glo has been shown to repress the translation of nuclear-encoded mitochondrial respiratory chain complex mRNAs (23). Glo also impacts ovarian nurse cell chromatin organization and dorsal-ventral patterning, possibly acting as a regulator of alternative splicing of *ovarian tumor* mRNA through its interaction with Hrp48 and Hfp (17,24). Most recently, Glo has been implicated in triglyceride homeostasis in adults by regulating mRNAs encoding lipid transport proteins (25). Finally, Glo is required for viability to adulthood (17), although the target RNAs and regulatory mechanism used in this capacity are not known.

How a single protein, Glo, regulates multiple mRNA metabolic pathways and how it distinguishes regulatory needs among its RNA targets remain largely elusive. One possibility is suggested by Glo's dual modes of RNA recognition. Glo contains 3 highly conserved quasi-RNA recognition motifs (qRRMs), each of which harbors 2 RNA-binding interfaces with distinct sequence specificities, allowing different combinations of RNA-binding modes to select subsets of target RNAs (18). Apart from the qRRMs, Glo contains no known protein domains. Thus, the qRRMs may also act as protein-protein interaction interfaces to recruit different effector proteins that diversify Glo's regulatory activities. To gain further insight into Glo as a multi-faceted translational repressor and into qRRM function beyond RNA binding, we isolated Glo-interacting proteins from late-stage oocytes and investigated whether the interaction is mediated by one or more of Glo's qRRMs.

Here, we identify the *Drosophila* fragile X mental retardation protein, dFMRP, as a Glo-interacting protein required for translational repression of *nos* mRNA during late stages of oogenesis. We show that dFMRP negatively regulates *nos* translation in late-stage oocytes and that dFMRP associates with polysome-bound *nos*, consistent with its proposed role as a translation elongation repressor. Using an *in vitro* translation run-off assay that distinguishes between effects on translation initiation and elongation, we show that Glo represses a reporter containing the *nos* TCE at both the initiation and elongation phases and that dFMRP specifically represses translation elongation. Puromycin release and [^35^S]-methionine incorporation assays demonstrated that dFMRP stalls ribosomes on *nos* and decreases elongation rate, respectively. Analysis of engineered Glo variants lacking individual qRRMs revealed that qRRM2 is specifically required for dFMRP binding to Glo and consequently to *nos in vivo*. Furthermore, an *in vitro* binding assay using purified qRRMs and dFMRP showed that qRRM2 interacts directly with dFMRP. Taken together, these results support a model in which Glo represses translation elongation of *nos* by directly recruiting dFMRP through qRRM2.

## MATERIALS AND METHODS

### Construction of transgenes and transgenic lines

The *gfp-glo* transgene was previously described (18). The transgene was maintained in flies homozygous for the *glo^162x^* null allele (17), so that GFP-Glo constituted the only source of Glo protein. This transgene fully rescues *glo^162x^* phenotypes (24). qRRM and CTX deletions were generated in the *glo* coding sequence by overlap extension PCR mutagenesis. All transgenes were inserted into the attP40 landing site by phiC31-mediated integration. *glo^162x^* mutant germline clones expressing *gfp-glo* or *gfp-gloΔqRRM* transgenes were generated using the dominant female-sterile method (26). The *gfp-dfmr1* transgenic line was previously described (27).

### Collection of ovary and oocyte samples

To obtain late-stage-oocyte-enriched ovaries (late-ovaries), female flies were fed for 4 days at 25°C in bottles of corn meal agar food supplemented with yeast paste and then starved in bottles with only wet Kimwipes (Kimtech) for 18 h at 25°C. Ovaries were hand-dissected on ice in PBS and used immediately to prepare extract. For late-stage oocyte isolation, hand-dissected ovaries were teased apart, and late-stage oocytes were separated out according to morphology, pooled in PBS, and used immediately. The *Oregon-R* strain was used for preparation of wild-type tissues. *dfmr1* RNAi tissues were produced by *mat-tub-Gal4* > *UAS-dfmr1RNAi* (TRiP GL0075) females.

### Immunoprecipitation and mass spectrometry (IP-MS)

Late-ovaries expressing GFP-tagged proteins were homogenized in IP buffer (10 mM Tris–HCl pH 7.5, 150 mM NaCl, 0.5 mM EDTA, 0.5% IGEPAL-CA630, 1x cOmplete EDTA-Free Protease Inhibitor Cocktail (Roche), 1 mM PMSF) supplemented with 20 μl of RNase A/T1 Mix (Thermo Scientific) and 20 U of RNase ONE (Promega) per 1 ml of IP buffer and cleared by centrifugation at 13,200 rpm for 30 min at 4°C. The supernatant was incubated with GFP-Trap_A beads (Chromotek) at 4°C for 1.5 h, and the beads were then washed with IP buffer without IGEPAL-CA630. Immunoprecipitates were eluted in 0.1% (v/v) TFA. For crosslinked IP-MS, dissected ovaries were incubated in PBS containing 1 mM DSP (Thermo Scientific) or in PBS without DSP (control) for 30 min at room temperature, quenched by addition of Tris–HCl pH 7.5 to a final concentration of 20 mM, and incubated for 15 min. Extract was prepared and immunoprecipitation with GFP-Trap_A beads was performed as described above, except that the beads were washed with IP buffer containing 1 M NaCl and no IGEPAL-CA630. Immunoprecipitates were eluted by boiling the beads in SDS-PAGE sample buffer (2% SDS, 62.5 mM Tris base, 10% glycerol, 100 mM DTT) which also cleaves the disulfide linker in DSP to reverse-crosslink. For both native and crosslinked IP-MS, LC–MS/MS was performed by the Proteomics and Mass Spectrometry Core at Princeton University. The resulting MS datasets were analyzed using Scaffold 4 (Proteome Software). Fold enrichment was defined as follows.

Native IP-MS:

$$\begin{equation*}Fold\ enrichment\ = \ \frac{{\# \ total\ spectral\ counts\ in\ {\boldsymbol{GFP}}\rm{\boldsymbol -}{\boldsymbol{Glo}}\ {\it IP}}}{{\# \ total\ spectral\ counts\ in\ {\boldsymbol{MCP}}\rm{\boldsymbol -}{\boldsymbol{GFP}}\ {\it IP}}}\end{equation*}$$

Crossliked IP-MS:

$$\begin{eqnarray*} && Fold\ enrichment \nonumber\\ && = \ \frac{{\# \ total\ spectra\ count\ in\ {\boldsymbol{crosslinked}}\ \left( { + \ DSP} \right)\ IP\ sample}}{{\# \ total\ spectra\ count\ in\ {\boldsymbol{un}} {\boldsymbol{crosslinked}}\ \left( { - \ DSP} \right)\ control}} \end{eqnarray*}$$

### Immunoprecipitation of endogenous Glo

10 μg of anti-Glo antibody (5B7) (17), or anti-GFP antibody (JL-8; Clontech) was crosslinked to 50 μl of Dynabeads Protein G (Thermo Fisher) by dimethyl pimelimidate. Wild-type *Drosophila* late-ovary lysate was prepared, incubated with beads, and washed as described above (native IP-MS), except that the IP buffer contained 200 mM NaCl.

### RNA co-immunoprecipitation

Late-ovary extracts were prepared as follows. 50 pairs of freshly dissected late-ovaries were homogenized on ice in 100 μl of RNA-IP buffer containing 10 mM HEPES–NaOH pH 7.0, 100 mM KCl, 5 mM MgCl_2_, 0.5% IGEPAL CA-630, 1 mM DTT, 200 U/ml SUPERase·In (Ambion), and 1× cOmplete EDTA-Free Protease Inhibitor Cocktail, nutated for 15 min, and cleared by centrifugation at 13,200 rpm for 30 min at 4°C. The supernatant was diluted in 900 μl of buffer containing 50 mM Tris–HCl pH 7.5, 150 mM NaCl, 20 mM EDTA, 1 mM DTT, 0.05% IGEPAL CA-630 and 200 U/ml SUPERase·In.

For RNA co-IP following polysome fractionation, RNP (fractions 1–5), monosomal (fractions 6–11), and polysomal (fractions 12–24) fractions were separately pooled. EDTA pH 8.0 was added to a final concentration of 50 mM. Diluted extract or pooled fractions were incubated overnight at 4°C with 75 μl of Dynabeads Protein G (Invitrogen) coupled to 5 μg of mouse anti-GFP monoclonal antibody (3E6; Invitrogen). After incubation, beads were washed 4 times in ice-cold wash buffer (50 mM Tris–HCl pH 7.5, 150 mM NaCl, 1 mM MgCl_2_ and 0.05% IGEPAL CA-630) and 2 times in wash buffer supplemented with 2 M urea. Washed beads were treated first with 100 μl of wash buffer containing 8 μl of RQ1 RNase-free DNase (Promega) for 15 min at 37°C and then with 150 μg/ml proteinase K (NEB) in the presence of 1% SDS for 30 min at 55°C. RNA was extracted with phenol:chloroform, precipitated with isopropanol, and re-dissolved in 20 μl of nuclease-free water.

### Immunoblotting

For analysis of Nos levels, late-ovary extract preparation and immunoblotting were performed as previously described (9) except that nitrocellulose membrane was used. For IP and co-IP experiments, immunoprecipitates were eluted by boiling the beads in SDS-PAGE sample buffer (2% SDS, 62.5 mM Tris base, 10% glycerol, 100 mM DTT), resolved on a 10% SDS-PAGE gel, and transferred to nitrocellulose membrane. The following primary antibodies were used: 1:1,000 rabbit anti-Nos (gift of A. Nakamura), 1:1,000 mouse anti-Glo (5B7) (17), 1:10,000 rabbit anti-Kinesin heavy chain (Khc; Cytoskeleton), 1:1,000 mouse anti-GFP (JL-8; Clontech), and 1:1,000 mouse anti-dFMRP (6A15; Abcam). Results were visualized by enhanced chemiluminescence (Roche) and autoradiographic film exposure. Relative Nos and Glo levels were quantified in ImageJ by normalizing band intensity to Khc.

### Polysome fractionation

Late-ovary extracts were prepared by homogenizing 200 pairs of freshly dissected late-stage-oocyte-enriched ovaries on ice in a lysis buffer containing 50 mM Tris–HCl pH 7.5, 250 mM NaCl, 50 mM Mg(OAc)_2_, 0.2% Triton X-100, 2 mg/ml heparin, 1 mM DTT, 200 U/ml SUPERase·In, 0.5 mg/ml cycloheximide (Sigma-Aldrich), and 1x cOmplete EDTA-Free Protease Inhibitor Cocktail. The extract was cleared by centrifugation at 13,200 rpm for 15 min at 4°C, and the supernatant was layered onto a 12 ml 10–50% sucrose gradient in 50 mM Tris–HCl pH 7.5, 250 mM NaCl and 50 mM Mg(OAc)_2_. Gradients were centrifuged in a Beckman SW41Ti rotor at 36,000 rpm for 3 h at 4°C. Following centrifugation, the gradient was fractionated using a Piston Gradient Fractionator (BioComp) to record UV absorbance at 260 nm and collect 500 μl fractions.

For RNA extraction, fractions were treated first with 10 μl of RQ1 RNase-free DNase for 30 min at 37°C and then with 150 μg/ml proteinase K in the presence of 1% SDS and 50 mM EDTA for 30 min at 55°C, followed by phenol:chloroform extraction and isopropanol precipitation, and re-dissolved in 20 μl of nuclease-free water.

### Puromycin release and polysome analysis

100 pairs of late-ovaries were dissected in PBS (–Puro) or PBS containing 3 mM puromycin (+Puro; Sigma-Aldrich) on ice. Dissected ovaries were transferred to 500 μl of lysis buffer (50 mM Tris–HCl pH 7.5, 250 mM NaCl, 5 mM Mg(OAc)_2_, 0.2% Triton X-100, 2 mg/ml heparin, 1 mM DTT, 200 U/ml SUPERase·In and 1× cOmplete EDTA-Free Protease Inhibitor Cocktail; –Puro) or lysis buffer containing 3 mM puromycin (+Puro) and incubated at 37°C for 15 min. Ovaries were then homogenized and incubated at 4°C for 15 min with constant mixing, incubated at 37°C for 10 min, and supplemented with cycloheximide (1 mg/ml final concentration) and Mg(OAc)_2_ (50 mM final concentration). The extract was cleared by centrifugation at 13,200 rpm for 15 min at 4°C, and the supernatant was layered onto a 12 ml, 10–50% sucrose gradient in 50 mM Tris–HCl pH 7.5, 250 mM NaCl, and 50 mM Mg(OAc)_2_. Gradients were centrifuged in a Beckman SW41Ti rotor at 36,000 rpm for 1.5 h at 4°C and were fractionated and RNA extracted as described in Polysome fractionation, except that 2 μl of 1 nM *Renilla* luciferase RNA was added to each fraction prior to RNA extraction as internal control for subsequent RT-qPCR analysis.

### RT-PCR

For analysis of RNA from immunoprecipitates and from sucrose gradient fractions, respectively, 1/2 and 1/10 of the total amount of RNA purified was reverse transcribed using SuperScript III Reverse Transcriptase (Invitrogen) with the following condition. Annealing: 1 μl of 50 μM oligo d(T)_20_ (Invitrogen), 1 μl of 10 mM dNTP Mix (Invitrogen), 2 μl (gradient fraction) or 10 μl (RNA co-IP) of extracted RNA was combined in a total volume of 13 μl, incubated at 65°C for 5 min, and chilled on ice for 2 min. First-strand cDNA synthesis: 4 μl of 5× First-Strand Buffer (Invitrogen), 1 μl of 0.1 M DTT, 1 μl of SUPERase·In, and 1 μl of SuperScript III RT (200 units/μl; Invitrogen) was added to a total volume of 20 μl, then incubated at 50°C for 50 min and treated with 1 μl of RNase H (NEB) at 37°C for 15 min. 2 μl of the RT products were PCR-amplified using standard Taq protocol with the following parameters. Annealing temperature: 54°C for *his3.3b* and 57°C for *nos* and *act5c*; extension time: 90 s for *his3.3b*, 60 s for *nos*, and 15 s for *act5c*; cycle number: 25 for *his3.3b* and *act5c* and 28 for *nos*. PCR products were resolved on a 1% (*his3.3b* and *nos*) or a 1.5% (*act5c*) agarose gel. For analysis of *nos* distribution in sucrose gradient, ethidium bromide-stained gels were imaged on an iBright FL1000 Imaging System (Thermo Fisher), and band intensity was quantified using the built-in analysis function. Band intensity of each fraction was normalized to the total intensity of all fractions, therefore the distribution result is independent of absolute transcript levels of *nos*. The following primers were used: *his3.3b*-F: 5′-dAAGGAGCACGGCGCAACGTAC-3′, *his3.3b*-R: 5′-dGATTGATTCCGCATAAAGCGCG-3′, *nos*-F: 5′-dGCGATCAAGGCGGAATCG-3′, *nos*-R: 5′-dATAGGATCCGAAAGTGTTCCTTGCTA, *act5c*-F: 5′-dAAGTACCCCATTGAGCACGG-3′, *act5c*-R: 5′-dACATACATGGCGGGTGTGTT-3′.

### RT-qPCR

For analysis of oocyte samples, RNA was extracted by homogenizing late-stage oocytes in TRIzol (Invitrogen) followed by chloroform extraction and isopropanol precipitation. 1 μg of total RNA was used for reverse transcription as described above. For both oocyte and gradient samples, 2 μl of cDNA was combined with 10 μl of 2× SYBR Green PCR Master Mix (Applied Biosystems) and 300 nM forward and reverse primers (final concentration) in a total volume of 20 μl. qPCR was performed on a StepOnePlus Real-Time PCR System (Applied Biosystems). Two (gradient samples) or three (oocyte samples) biological replicates were analyzed with three technical replicates each, all using auto thresholding and the following parameters: Holding, 95°C for 10 min; Cycling, 95°C for 15 s, 60°C for 1 min, repeated for 39 cycles; Melt curve, 95°C for 15 s, 60°C for 1 min, + 0.3°C step & hold, 95°C for 15 s. *rpl32* and *rluc* were used as internal control for oocyte and gradient samples, respectively, unless otherwise stated. Relative levels of other target transcripts were calculated as follows (Ct of un-detected sample was set as 40).

$$\begin{equation*}Relative\ level\ = \ {2^{\left( {C{t_{target\ transcript}}\ -\ C{t_{internal\ control}}} \right)}}\end{equation*}$$

For polysome analysis and 80S formation analysis,

$$\begin{eqnarray*} && Fractional\ transcript\ level \nonumber\\ && = {{{relative\ transcript\ level}} \!{\left/ {\vphantom {{relative\ transcript\ level} {\mathop \sum \ relative\ transcript\ level}}}\right.} \!{{\mathop \sum \nolimits relative\ transcript\ level}}}\ \end{eqnarray*}$$

The following primers were used: *rpl32*-F: 5′-dCGGATCGATATGCTAAGCTGT-3′, *rpl32*-R: 5′-dGCGCTTGTTCGATCCGTA-3′, *nos*-qPCR-F: 5′-dCACCGCCAATTCGCTCCTTAT-3′, *nos*-qPCR-R: 5′-dGCTGGTGACTCGCACTAGC-3′, *act5c*-F: 5′-dAAGTACCCCATTGAGCACGG-3′, *act5c*-R: 5′-dACATACATGGCGGGTGTGTT-3′, *ha-fluc*-qPCR-F: 5′-dCCCCGGGAAGACGCCAAAAACATAAAG-3′, *ha-fluc*-qPCR-R: 5′-dCGTATCTCTTCATAGCCT-3′, *rluc*-qPCR-F: 5′-dGATAACTGGTCCGCAGTGGT-3′, *rluc*-qPCR-R: 5′-dACCAGATTTGCCTGATTTGC-3′.

### Generation of reporter and competitor RNAs for *in vitro* translation assay

The luciferase reporter RNAs were constructed in a derivative of a BSK-A vector containing the firefly luciferase coding sequence followed by 73 adenosine residues (kindly provided by F. Gebauer and M. W. Hentze). For firefly luciferase (Fluc) reporters, *nos* 5′ UTR sequence was sub-cloned from the pA25-nos5′UTR plasmid (9) and inserted between the SacI and SmaI sites. 3xTCE, 3xTCEIIIA, 3xSRE^–^ and *tub* 3′ UTR sequences were sub-cloned from respective plasmids (9) and inserted between the BglII and BamHI sites. For the *Renilla* luciferase (Rluc) reporter, the SmaI-BamHI fragment containing the firefly luciferase coding sequence in the BSK-A derivative plasmid was replaced by the *Renilla* luciferase coding sequence. HA-tagged Fluc reporters were generated as follows. The *nos* 5′UTR sequence was sub-cloned from the pA25-nos5′UTR plasmid (9) and inserted between the SacI and NcoI sites of a FLAG-3xHA vector (kindly provided by T. Aoki and P. Schedl). Firefly luciferase coding sequence with 3xTCE or 3xTCEIIIA sequences followed by 73 adenosine residues was sub-cloned from the respective Fluc reporter plasmids (described above) and inserted between the SmaI and HindIII sites. Capped reporter RNA was transcribed from 1 μg of HindIII-linearized plasmid DNA using the mMessage mMachine T3 Transcription kit (Ambion). TCEII and TCEIII competitor RNAs were generated from BamHI-linearized plasmids containing individual TCE stem-loop II and TCE stem-loop III sequences (17), respectively, using the MEGAscript T7 Transcription kit (Ambion). In all cases, *in vitro* transcription reactions were treated with 1 μl of TURBO DNase (Ambion) for 15 min at 37°C, and RNA was extracted with phenol:chloroform and precipitated first with isopropanol and then with ethanol. Prior to use, the competitor RNA was de-natured at 65°C for 5 min and slowly cooled to room temperature (17).

### *In vitro* translation and translation run-off assay

Late-ovary translation extract was prepared as previously described (20) except that 1× cOmplete EDTA-Free Protease Inhibitor Cocktail was used in place of 0.5 mM PMSF. Extract was made, aliquoted, flash frozen in liquid N_2_, and stored at –80°C for no more than 2 weeks before use.

The *in vitro* translation reaction (20 μl) contained 50% late-ovary translation extract, 1× translation mix (25 mM HEPES–NaOH pH 7.5, 3 mM Mg(OAc)_2_, 2.5 mM DTT, 25 μM Complete Amino Acid Mixtures (Promega), 1.2 mM ATP, and 0.3 mM GTP), energy regeneration system (15 mM creatine phosphate and 0.1 mg/ml creatine phosphokinase (Sigma-Aldrich)), 0.1 nM *Renilla* luciferase reporter RNA (internal control), and 1 nM firefly luciferase reporter RNA. Reactions were incubated at 28°C for 2 h. Additional external control reactions (25 μl) were assembled as follows. The same firefly-*Renilla* luciferase reporter pair (0.1 nM each, final concentration) was incubated in 17.5 μl of Nuclease-Treated Rabbit Reticulocyte Lysate (Promega), 40 μM Complete Amino Acid Mixtures, and 1 μl of Human Placenta RNase Inhibitor (NEB). The reaction was allowed to proceed for 90 min at 28°C. Luciferase activity was assayed using the Dual-Luciferase Reporter Assay System (Promega), and luminescence was measured by a Glomax 20/20 Luminometer (Promega) using the built-in DLR protocol. The relative reporter activity and fold repression were defined as follows.

$$\begin{eqnarray*} && Relative\ reporter\ activity \nonumber\\ && = \ \frac{{{{[{{{Fluc\ activity}} \!\mathord{\left/ {\vphantom {{Fluc\ activity} {Rluc\ activity}}}\right.} \!{{Rluc\ activity}}}]}_{ovary\ lysate}}}}{{{{[{{{Fluc\ activity}} \!\mathord{\left/ {\vphantom {{Fluc\ activity} {Rluc\ activity}}}\right.} \!{{Rluc\ activity}}}]}_{rabbit\ reticulocyte\ lysate}}}}\end{eqnarray*}$$

$$\begin{equation*}Normalized\ reporter\ activity\ = \ \frac{{{{[relative\ reporter\ activity]}_{any\ 3^{\prime}UTR\ }}}}{{{{[relative\ reporter\ activity]}_{tub3^{\prime}UTR}}}}\end{equation*}$$

$$\begin{equation*}Fold\ repression\ = \ {{1} \!\mathord{\left/ {\vphantom {1 {Normalized\ reporter\ activity}}}\right.} \!{{Normalized\ reporter\ activity}}}\end{equation*}$$

Data were averaged over three biological replicates using different preparations of extract.

For the translation run-off assay, the reaction mixture was assembled without the energy regeneration system, pre-incubated at 16°C for 5 min. Either 0.1 μl of 10 mg/ml 4E1RCat (in DMSO; Sigma-Aldrich) (the final concentration of 4E1RCat is 50 μg/ml which effectively blocks 80S ribosome formation as determined by the 80S formation assay below) or 0.1 μl of DMSO was added, followed by 1 μl of TCEII or TCEIII competitor RNA (2 μg/μl) or nuclease-free water. Finally, the energy regeneration system was added, and the reaction was incubated at 28°C for 2 h. Fold repression was calculated as described above except that the value was normalized to the value of 3xTCEIIIA without 4E1RCat or competitor RNA treatment.

$$\begin{eqnarray*} && Elongation\ repression \nonumber\\ && = \ {{{{{\left[ {fold\ repression} \right]}_{TCE}}}} \!\mathord{\left/ {\vphantom {{{{\left[ {fold\ repression} \right]}_{TCE}}} {{{\left[ {fold\ repression} \right]}_{TCE + 4E1RCat + TCEIII}}}}}\right.} \!{{{{\left[ {fold\ repression} \right]}_{TCE + 4E1RCat + TCEIII}}}}}\end{eqnarray*}$$

$$\begin{eqnarray*} && Initiation\ repression \nonumber\\ && = \ {{{{{\left[ {fold\ repression} \right]}_{TCE + 4E1RCat + TCEIII}}}} \!\mathord{\left/ {\vphantom {{{{\left[ {fold\ repression} \right]}_{TCE + 4E1RCat + TCEIII}}} {{{\left[ {fold\ repression} \right]}_{TCE + TCEIII}}}}}\right.} \!{{{{\left[ {fold\ repression} \right]}_{TCE + TCEIII}}}}}\end{eqnarray*}$$

The data were averaged over three biological replicates using different preparations of extract.

### 80S formation assay

10 μl of 10 nM Rluc reporter was incubated in a total volume of 100 μl of *in vitro* translation reaction mixture containing 50% *Drosophila* ovary translation extract (as described above) in the presence of 1 mg/ml cycloheximide. 0.5 μl of 10 mg/ml 4E1RCat (+4E1RCat) or DMSO (–4E1RCat) was added, and the mixture was incubated at 25°C for 30 min followed by polysome fractionation. 2 μl of 1 nM HA-Fluc reporter RNA was added to each fraction as an internal control for subsequent RT-qPCR analysis, and RNA from each fraction was prepared as described in Puromycin release and polysome analysis.

### [^35^S]-Methionine incorporation assay

Reactions containing 50 μl of *Drosophila* late-ovary extract, 10 μl of 10× translation mix (250 mM HEPES–NaOH pH 7.5, 30 mM Mg(OAc)_2_, 25 mM DTT, 250 μM Amino Acids Mixture Minus Methionine (Promega), 12 mM ATP and 3mM GTP), 10 μl of 5 nM *HA-Fluc* reporter RNA, 2.5 μl of 190 μM methionine (Sigma-Aldrich), 25 μCi [^35^S]-methionine (2.5 μl of ∼10 μM equivalent; PerkinElmer NEG709A001MC), and 10 μl of nuclease-free water were pre-incubated at 25°C for 10 min. The concentration of unlabelled methionine was optimized to sustain efficient elongation while retaining sufficient [^35^S]-Met incorporation. 10% of initial volume was removed at 0 and 5 min for anti-HA IP and scintillation counting. At 10 min, 2.5 μl of 10 mg/ml 4E1RCat, 15 μl of 100 mM creatine phosphate, and 2 μl of 5 mg/ml creatine phosphokinase were added to the reaction mixture to block *de novo* initiation and start translation run-off. For experiments involving addition of dFMRP or BSA, 10 μl of 1 mg/ml purified ΔNT-dFMRP (see Protein expression and purification below) or 1 mg/ml BSA (Sigma-Aldrich) was also added to the reaction mixture at 10 min. Additional aliquots equivalent to 10% of initial volume were removed at 10, 15, 20, 30, 45, 60, 90 and 120 min for anti-HA IP and scintillation counting. For anti-HA IP, aliquots were incubated with 5 μl of Pierce Anti-HA Magnetic Beads (Thermo Fisher) in 250 μl of RIPA buffer (25 mM Tris–HCl pH 7.5, 150 mM NaCl, 1% IGEPAL CA-630, 1% sodium deoxycholate (Sigma-Aldrich) and 0.1% SDS) containing 1 mM methionine at room temperature for 1 h with constant mixing. The beads were washed three times in 500 μl of TBST containing 0.1% SDS, re-suspended twice with 500 μl of UniverSol-ES Liquid Scintillation Cocktail (MP Biomedicals), and radioactivity was quantified by liquid scintillation counting in a TRI-CARB 4810TR Liquid Scintillation Counter (PerkinElmer) using the built-in S-35 counting protocol. Values for each time-point were averaged over two (+dFMRP and +BSA) or three (all others) biological replicates using different preparations of extract and were normalized to the values for the TCEIIIA reporter at 120 min.

### Protein expression and purification

The pET15b plasmids encoding N-terminal hexahistidine (His_6_)-tagged qRRM domains of Glo (qRRM1, residues 45–141; qRRM2, residues 142–234; qRRM3, residues 475–562) were previously described (18). The ΔNT-dFMRP (residues 220–681) coding sequence was sub-cloned from a pTYB1 NT-dFMRP construct (28) into pET15b (Novagen) with N-terminal His_6_-tag. Individual qRRM domains and ΔNT-dFMRP were expressed in *E. coli* strain Rosetta (DE3)-pLysS (Novagen).

For purification of individual qRRM domains, *E. coli* cultures were grown in LB medium to an OD_600_ of 0.6–0.7 and induced overnight with 0.5 mM IPTG at 16°C. The cells were pelleted, resuspended in buffer containing 50 mM Tris–HCl pH 8.0, 500 mM NaCl and 2 mM PMSF, and lysed using a cell disrupter. Cell lysate was cleared by centrifugation and the qRRM proteins were purified by Ni-NTA agarose (Qiagen) chromatography. Eluates were further purified by size-exclusion chromatography (Superdex 200 Increase 10/300, GE Healthcare), concentrated, flash-frozen, and stored at –80°C in 20 mM HEPES–NaOH pH 8.0 and 200 mM NaCl (qRRM1 and qRRM3) or 20 mM HEPES–NaOH pH 8.0 and 250 mM NaCl (qRRM2).

For purification of ΔNT-dFMRP, *E. coli* culture was grown in LB medium to an OD_600_ of 0.4–0.5 and induced overnight with 0.5 mM IPTG at 16°C. The cells were pelleted, re-suspended in buffer containing 50 mM Tris–HCl pH 8.0, 500 mM NaCl, 20 mM imidazole, 0.1% CHAPS, 5 mM β-mercaptoethanol, and 2 mM PMSF. The cells were lysed using a cell disruptor, and the lysate was cleared by centrifugation and applied to Ni-NTA agarose chromatography. The eluted protein was further purified by size-exclusion chromatography (Superdex 200 Increase 10/300), concentrated, flash-frozen, and stored at –80°C in 20 mM HEPES–NaOH pH 8.0, 250 mM NaCl and 2 mM DTT.

### His_6_-tag pull-down assay

Purified individual qRRMs (50 μg each) were immobilized on Ni-NTA agarose beads separately, washed in 20 mM Tris–HCl pH 7.5, 500 mM NaCl and 0.5% IGEPAL CA-630, and incubated with late-ovary extract (prepared as described in the IP-MS section) expressing GFP-dFMRP for 1.5 h at 4°C. The beads were washed three times with IP buffer (above) and eluted in boiling SDS-PAGE sample buffer. The IP products were resolved on a 15% SDS-PAGE gel, transferred onto nitrocellulose membrane, and detected by Ponceau S staining (qRRMs) or anti-GFP immunoblotting (GFP-dFMRP).

### Analytical size-exclusion chromatography binding assay

Proteins were diluted to 2.5 μM (ΔNT-dFMRP) or 20 μM (individual qRRM domains) in 20 mM HEPES–NaOH pH 8.0, 150 mM NaCl, and 5 mM TCEP in a volume of 60 μl, incubated at 4°C for 48 h, and then loaded onto a Superdex 200 Increase 3.2/300 column. The fractions were collected, and the proteins were concentrated by TCA-precipitation, resolved on a 15% SDS-PAGE gel, and visualized by staining with Coomassie Brilliant Blue R-250.

### Statistics

All statistical analyses were performed in GraphPad Prism 9.0.2. A two-tailed Welch *t*-test was used for comparisons of two means, and post hoc Holm-Sidak test was used for multiple comparison correction when necessary. Brown-Forsythe and Welch one-way ANOVA with post hoc Dunnett T3 test was used for one-dimensional comparisons of more than two means. Two-way full-model ANOVA with post hoc Tukey test was used for two-dimensional comparisons of means within and between genotype groups. Linear regressions of [^35^S]-methionine incorporation data were performed using the Simple Linear Regression function which also tested the significance of the difference between slopes. Data were plotted as mean or mean ± SEM from two or three biological replicates as indicated in the figure legends. Individual data points are shown unless for visibility reasons.

## RESULTS

### Identification of dFMRP as an RNA-independent Glo-interacting protein

**Figure 1.**
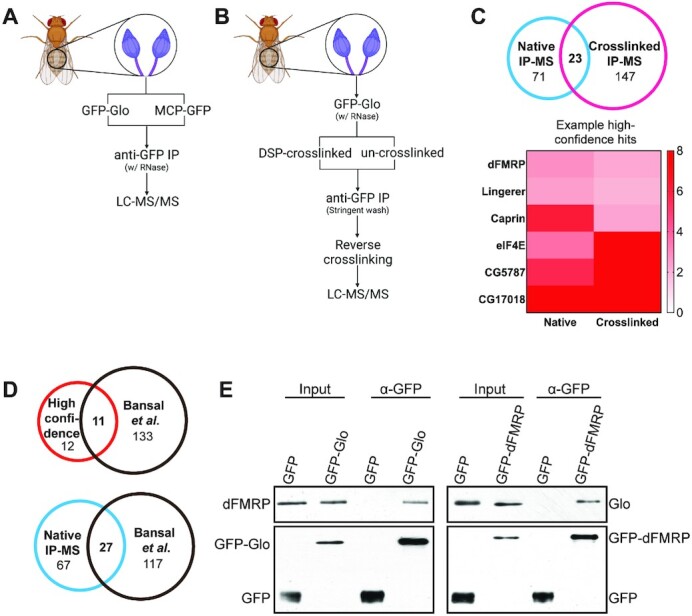
Identification of dFMRP as a RNA-independent Glo-interacting protein by IP-MS. (**A**) Schematic representation of the native IP-MS experiment. The total peptide count in GFP-Glo immunoprecipitates was compared to that in MCP-GFP immunoprecipitates. (**B**) Schematic representation of the crosslinked IP-MS experiment. The total peptide count in DSP-crosslinked immunoprecipitates was compared to that in un-crosslinked immunoprecipitates. (**C**) Top: Venn diagrams comparing native and crosslinked IP-MS results from (A, B). Bottom: Heat map showing examples of high-confidence IP-MS interactors. Heat-map scale indicates fold enrichment. (**D**) Venn diagrams comparing the >2-fold enriched set of IP-MS interactors identified in the Bansal dataset (34) with our >2-fold enriched native IP-MS interactors (middle) or with our pooled high-confidence interactors (bottom). (**E**) Immunoblots of extract prior to anti-GFP immunoprecipitation (input lanes) and anti-GFP immunoprecipitates (α-GFP lanes) from late-ovaries expressing GFP, GFP-Glo or GFP-dFMRP probed with anti-dFMRP antibody (top left panel), anti-Glo antibody (top right panel), or anti-GFP antibody (bottom panels). Endogenous dFMRP co-immunoprecipitated with GFP-Glo, and endogenous Glo co-immunoprecipitated with GFP-dFMRP.

To understand how Glo – which contains only annotated RNA-binding domains – represses *nos* translation, we isolated Glo interacting proteins from ovaries enriched for late-stage oocytes (late-ovaries) (20) by immunoprecipitation coupled to mass spectrometry (IP-MS). Anti-GFP immunoprecipitation was performed using late-ovaries expressing functional GFP-tagged Glo (GFP-Glo) in the absence of endogenous, native Glo. Late-ovaries expressing GFP-tagged bacteriophage MS2 coat protein (MCP-GFP) were used as a control (Figure 1A). Ovary extracts were treated with RNase prior to immunoprecipitation to eliminate RNA-dependent protein interactions. Mass spectrometry (MS) analysis identified 94 proteins, including Glo itself, that were at least 2-fold enriched in GFP-Glo immunoprecipitates relative to MCP-GFP immunoprecipitates (Supplementary Table S1). To facilitate recovery of potentially weak and transient Glo-interactors, we also performed crosslinking-based IP-MS under stringent conditions (Figure 1B) and detected 170 proteins that were at least 2-fold enriched in the GFP-Glo immunoprecipitates from the crosslinked sample compared to those from the un-crosslinked control (Supplementary Table S1). A single experiment was performed for each condition. 23 interactors were common to both the native and the crosslinked IP-MS experiments and were therefore considered high-confidence Glo-interacting proteins (Figure 1C; Supplementary Table S1). Among the high-confidence interactors are eIF4E and ribosomal subunits, consistent with Glo's implicated functions in regulating translation initiation and elongation of *nos*, respectively. The list also contains known translation regulators, including dFMRP (29,30), Caprin (31) and Lingerer (32,33), consistent with the function of Glo as a translational repressor more generally. Our high-confidence interactors (11 out of 23) overlap those identified in a recently reported Glo native IP-MS experiment using non-staged ovaries (34) (Figure 1D; Supplementary Table S1), further supporting the validity of the interactors identified here.

We focused on dFMRP because studies of dFMRP and its mammalian homologs have identified multiple modes of action in translational control, including inhibition of translation initiation (35,36) and ribosome stalling (28,37). Interaction of Glo and dFMRP in late-ovaries was further tested by reciprocal *in vivo* co-immunoprecipitation (co-IP) in the presence of RNase. Endogenous dFMRP and Glo were detected in anti-GFP immunoprecipitates from late-ovaries expressing GFP-dFMRP or GFP-Glo, respectively, but were not detected in control immunoprecipitates (Figure 1E). Endogenous dFMRP also co-immunoprecipitated with endogenous Glo (Supplementary Figure S1), confirming that dFMRP is an RNA-independent Glo-interacting protein during late oogenesis.

### dFMRP negatively regulates Nos protein levels

**Figure 2.**
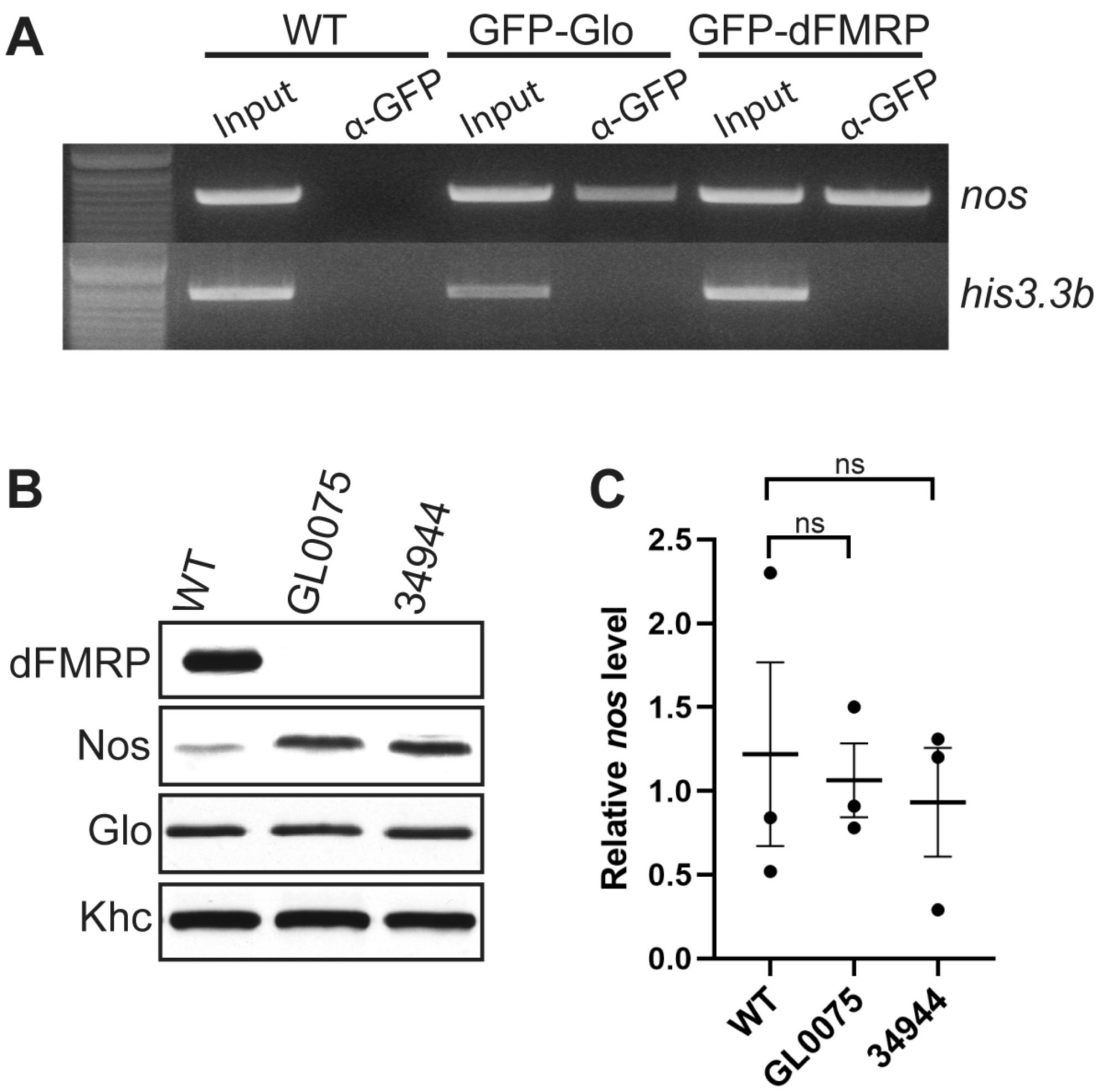
dFMRP negatively regulates Nos levels. (**A**) Ethidium bromide-stained gel analysis of RT-PCR for *nos* and control *his3.3b* RNA in anti-GFP immunoprecipitates from wild-type (WT), GFP-Glo expressing, and GFP-dFMRP expressing late-ovaries. *nos*, but not *his3.3b* RNA co-immunoprecipitated with Glo and dFMRP and was not detected in the control WT IP. (**B**) Immunoblots detecting dFMRP, Nos, Glo, and Khc (loading control) in wild-type (WT) and dFMRP KD (TRiP GL0075 and TRiP 34944 RNAi lines) in hand-dissected stage 13/14 oocytes. Both RNAi lines effectively reduced dFMRP expression. Nos level relative to wild-type: 4.3-fold in GL0075 and 4.5-fold in 34944; Glo level relative to wild-type: 1.1-fold in GL0075 and 1.2-fold in 34944. (**C**) Relative quantification of *nos* RNA levels in wild-type and dFMRP KD late-stage oocytes by quantitative RT-PCR. *dfmr1* RNAi did not impact *nos* RNA level in late-stage oocytes. Data are plotted as mean ± SEM from 3 biological replicates each with three technical replicates; *P* > 0.05 (ns) by Brown-Forsythe and Welch one-way ANOVA with post hoc Dunnett T3 test.

We next sought to determine whether dFMRP, like Glo, participates in *nos* regulation. To test whether dFMRP interacts with *nos* mRNA *in vivo*, we performed RNA co-IP experiments using wild-type, GFP-Glo, or GFP-dFMRP late-ovaries. *nos* could be detected by RT-PCR in both GFP-Glo and GFP-dFMRP immunoprecipitates, whereas a control maternal transcript, *his3.3b*, was not detected (Figure 2A). To probe a requirement for dFMRP in translational repression of *nos*, we knocked down *dfmr1* expression in the female germline. Expression of either of two independent *UAS-dfmr1* RNAi transgenes with a *maternal-tubulin-Gal4* driver resulted in almost complete knock-down (KD) of dFMRP protein expression (un-detectable by western blotting, Figure 2B). For both RNAi lines, Nos protein levels were increased by ∼4.5-fold in late-stage ovaries (Figure 2B) as compared to wild-type. This up-regulation was not a result of a decrease in the amount of Glo protein (Figure 2B) or an increase in *nos* mRNA levels (Figure 2C). These results suggest that dFMRP interacts with *nos* to negatively regulate its translation during late oogenesis. Since the *dfmr1* RNAi lines behaved similarly, we used the TRiP GL0075 line in all subsequent experiments requiring dFMRP KD.

### dFMRP reduces ribosome load on *nos*

**Figure 3.**
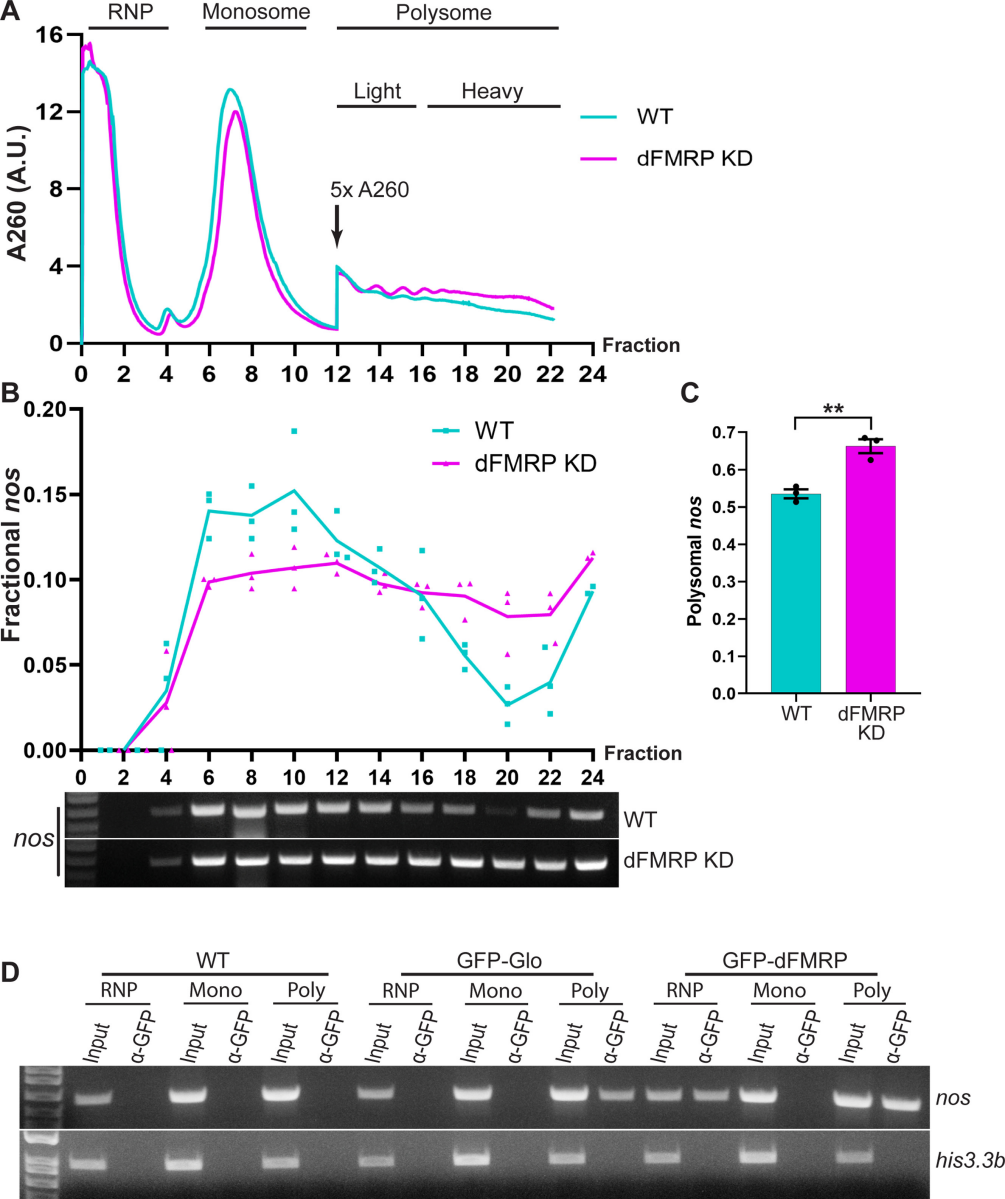
dFMRP KD increases polysome association of *nos*. (**A**) UV_260_ absorbance profile from representative 10–50% sucrose density gradients. *A*_260_ values are shown at 5-fold in polysome fractions for visualization purposes. Light polysomes (fractions 12–15) contains 2–4 ribosomes per transcript; heavy polysomes (fractions 16–24) contains five or more ribosomes per transcript. (**B**) Analysis of *nos* RNA distribution in sucrose gradient fractions. Quantification of relative *nos* levels in gradient fractions from wild-type (WT) and dFMRP KD late-ovaries (top) as determined by RT-PCR (bottom). Individual data points are plotted and their mean values (*n* = 3) are shown by trend lines. *nos* levels were calculated as a fraction of total *nos* band intensity. Representative ethidium bromide-stained agarose gels of *nos* RT-PCR products are shown. (**C**) Fraction of *nos* RNA associated with polysomes in WT (54%) and dFMRP KD (66%) late-ovaries. Values are mean ± SEM from three biological replicates; **P < 0.01 by two-tailed Welch *t*-test. (**D**) RT-PCR analysis of RNA isolated following anti-GFP IP of sucrose gradient fractions shown in (A) for WT, GFP-Glo expressing, and GFP-dFMRP expressing late-ovaries: RNP (RNP, fractions 1–5), monosomal (Mono, fractions 6–11), and polysomal (Poly, fractions 12–24) fractions. GFP-dFMRP co-immunoprecipitated with *nos*, but not *his3.3b*, RNA in both RNP and polysomal fractions.

To determine how dFMRP regulates *nos* translation, we first performed sucrose density gradient sedimentation to monitor ribosome load on *nos* in wild-type and in dFMRP KD late-ovaries. We hypothesized that if dFMRP only affects translation initiation of *nos* mRNA, dFMRP KD would increase polysome association of *nos* but leave its distribution within the polysomal fractions unchanged. In contrast, if dFMRP plays a role in regulating translation elongation, dFMRP KD would affect the number of ribosomes on *nos* mRNA, hence the relative level of *nos* in light (fewer ribosomes) versus heavy (more ribosomes) polysome fractions. Consistent with previous studies (22,38), the overall polysome signal was low (Figure 3A), presumably due to generally low translational activity during the later stages of oogenesis. While the overall polysome profile did not show an obvious change upon dFMRP KD, there was a re-distribution of *nos* mRNA toward the heavier fractions (Figure 3B). Specifically, the shift of *nos* mRNA from lighter to heavier polysome fractions suggests that dFMRP regulates translation elongation of *nos* mRNA. In addition, we observed a moderate increase in the percentage of polysome-associated *nos*, from ∼50% in wild-type, which is consistent with our previous findings (20,22), to ∼65% in dFMRP KD late-ovaries (Figure 3C), indicating either that dFMRP also represses *nos* translation initiation or that initiation-based repression plays a secondary role and, by itself, does not completely eliminate ribosome loading onto *nos* mRNA. Together with the results presented above (Figure 2), these data provide further evidence that dFMRP is a translational repressor of *nos*.

### dFMRP represses translation elongation of *nos* through the TCE

**Figure 4.**
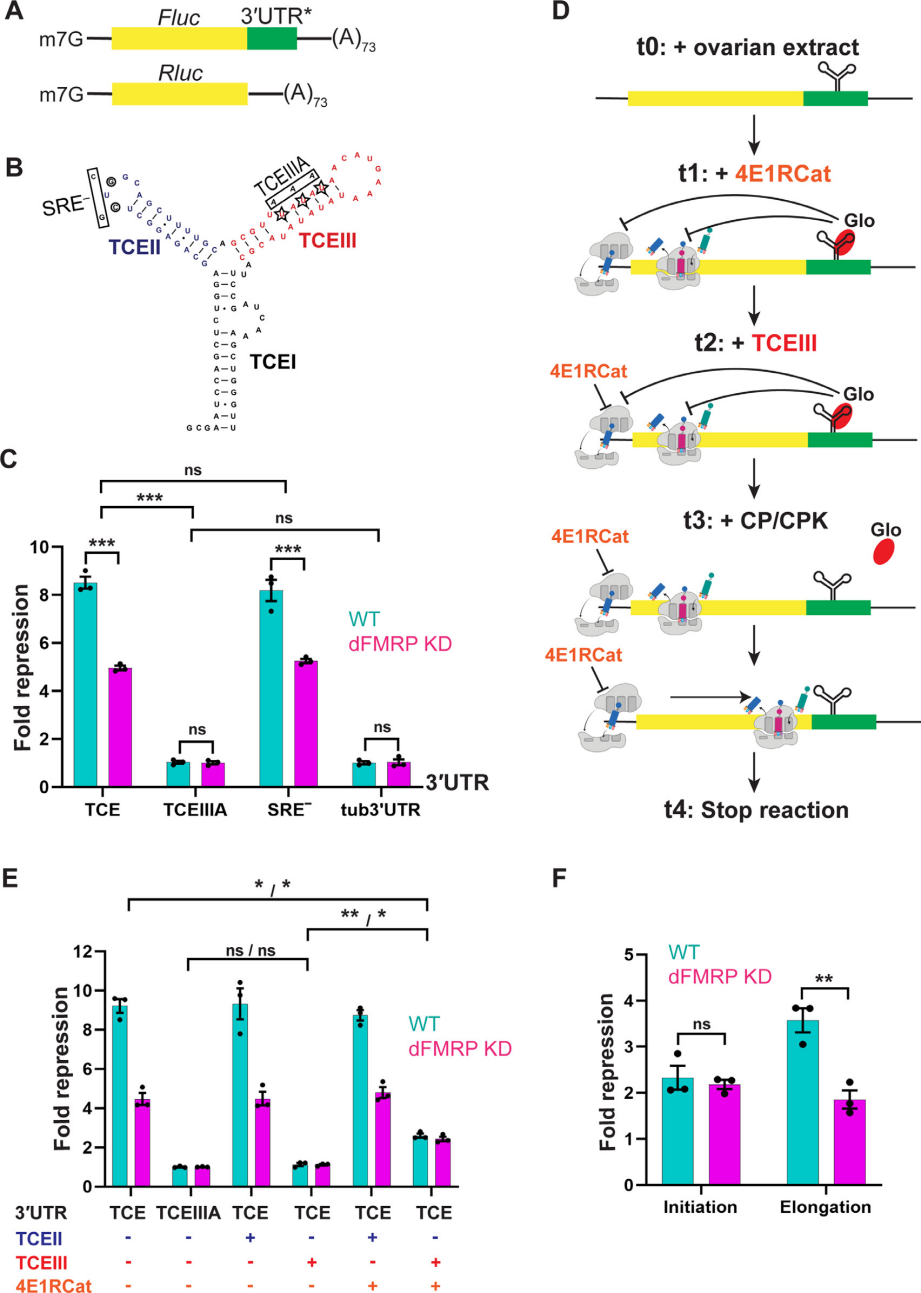
Biochemical dissection of TCE-mediated *nos* regulation. (**A**) Schematics of the reporters used. The firefly luciferase (*FLuc*) coding sequence was fused to either 3xTCE (TCE), 3xTCEIIIA (TCEIIIA), 3xTCE[SRE^–^] (SRE^–^), or *α-tubulin* 3′ UTR (tub3′UTR) sequences, as indicated by 3′UTR*. The *Renilla* luciferase coding sequence did not contain specific 3′ UTR sequence and was not subject to translational regulation. Both reporters contained a 5′ 7-methylated guanosine (m^7^G) cap and 3′ poly (A)_73_ tail. (**B**) Schematic representation of the *nos* TCE. Glo binds to both TCEI and TCEIII, whereas Smg binds to TCEII. The TCEIIIA and SRE^–^ mutations disrupt Glo and Smg binding, respectively. (**C**) *In vitro* translation assay using reporters shown in (A). For each indicated reporter, fold repression is the inverse of the normalized relative luciferase activity, which was calculated as the ratio of firefly to *Renilla* luciferase activity in late-ovary extract normalized first to activity in rabbit reticulocyte lysate and then to the value for the *tub3*′*UTR* reporter. (**D**) Experimental setup of the translation run-off assay. Reporter RNAs were pre-incubated in late-ovary extract without the energy regeneration system (t_0_). The translation initiation inhibitor 4E1RCat (50 μg/ml) was then added to block *de novo* initiation (t_1_), followed by the addition of competitor RNAs (t_2_; control TCEII or specific TCEIII). Finally, the energy regeneration system was added back to restore translation (t_3_), and luciferase activity was assayed (t_4_). (**E**) Results of the translation run-off assay in wild-type (WT) and dFMRP KD ovary extracts. Normalized relative luciferase activity and fold repression were calculated as in (C), except that the luciferase activity was normalized to the value for TCEIIIA (without 4E1RCat or competitor RNA). (**F**) Contributions of translation initiation and elongation to fold repression. [Fold repression]_elongation_ = [Fold repression]_TCE_/[Fold repression]_TCE+4E1RCat+TCEIII_. [Fold repression]_initiation_ = [Fold repression]_TCE+4E1RCat+TCEIII_/[Fold repression]_TCE+TCEIII_. In (C), (E) and (F), values are mean ± SEM from three biological replicates from different ovary extract preparations. Statistical significance was analyzed using two-way full-model ANOVA with post hoc Tukey test (in C), Brown-Forsythe and Welch one-way ANOVA with post hoc Dunnett T3 test (in E, significance symbol for wild-type/dFMRP KD), and two-tailed Welch *t*-test with post hoc Holm-Sidak test (in F); *P < 0.05, **P < 0.01 and ***P < 0.001, not all tested *P*-values are shown.

To determine more definitively if dFMRP participates in one or both modes of TCE/Glo-mediated *nos* repression, we optimized a previously established *in vitro* translation system (9,20) based on late-ovary extract that confers TCE-mediated repression on a reporter RNA and performed a translation run-off assay. First, we compared the ability of extracts from wild-type and dFMRP KD late-ovaries to support TCE-mediated repression of a firefly luciferase (*Fluc*) reporter RNA. Extracts were programmed with capped and polyadenylated *Fluc* fused to either 3 tandem copies of the wild-type *nos* TCE (*Fluc-3xTCE*) or the unregulated *α-tubulin* (*tub*) 3′ UTR (*Fluc-tub3*′*UTR*), along with *Renilla* luciferase (*Rluc*) RNA that served as an internal control for experimental variability (Figure 4A, B). In the wild-type extract, *Fluc-3xTCE* RNA was repressed 9-fold compared to the unregulated *Fluc-tub3*′*UTR* RNA (Figure 4C). By contrast, only 4.5-fold repression of *Fluc-3xTCE* RNA was observed for the dFMRP KD extract (Figure 4C). As we showed previously, the TCEIIIA mutation, which disrupts Glo binding and *nos* repression *in vivo* (9,17), completely abolished repression of the reporter RNA in the wild-type extract (*Fluc-3xTCEIIIA*), whereas repression was not affected by mutation of the Smg-binding site in TCEII (*Fluc-3xSRE^–^*) (Figure 4B, C). Similarly, the TCEIIIA mutation eliminated repression of the reporter in the dFMRP KD extract, whereas the SRE^–^ mutation had no effect (Figure 4C). These results indicate that the *in vitro* translation system recapitulatea *nos* regulation *in vivo* and that dFMRP accounts in part for Glo/TCE-mediated *nos* translational repression.

Next, we performed a translation run-off assay to biochemically dissect *nos* regulation at the translation initiation and elongation phases (Figure 4D, also see Materials and Methods). Reporter RNA was first incubated in late-ovary extract without the ATP/GTP regeneration system to allow the assembly of the repression complex and the initial loading of ribosomes (39). Subsequently, the translation initiation inhibitor 4E1RCat, which competitively disrupts the interaction of eIF4G with eIF4E and hence the formation of the eIF4F complex (40), was added to block *de novo* initiation on the reporter RNA. 4E1RCat reduced the absolute expression level of luciferase reporters in a dosage-dependent manner (Supplementary Figure S2A) but did not affect TCE-mediated translational repression (Supplementary Figure S2B). Sucrose gradient analysis confirmed that 4E1RCat effectively prevented 80S ribosome formation on the reporter RNA in late-ovary extract (Supplementary Figure S2C, D). Competitor TCEIII RNA was then added to titrate the TCE-bound repression complex from the reporter RNA. Finally, the ATP/GTP regeneration system was provided to resume translation. In this way, we were able to directly test if the *nos* TCE imposes blocks at both translation initiation and elongation, manifested by partial de-repression by TCEIII in the presence of 4E1RCat, and to de-couple the two repression modes to assess the impact of dFMRP on each independently.

As expected, addition of TCEIII competitor RNA to the wild-type extract almost fully relieved repression of the *Fluc-3xTCE* reporter (1.1-fold repression for *Fluc-3xTCE* + TCEIII versus 9.0-fold for *Fluc-3xTCE*), whereas a control competitor, TCEII RNA, had no effect as expected (20) (Figure 4E). In the presence of 4E1RCat, addition of the TCEIII competitor, but not TCEII, partially de-repressed *Fluc-3xTCE* RNA (2.6-fold repression for *Fluc-3xTCE* + TCEIII + 4E1RCat versus 9.0-fold for *Fluc-3xTCE*, 3.6-fold up-regulated; Figure 4E). Since *de novo* initiation was inhibited, the 3.6-fold increase in reporter activity reflects the elimination of a TCE-mediated elongation block. Maximal de-repression was observed only when initiation was allowed to occur (-4E1RCat), however, indicating that full de-repression resulted from elimination of both an initiation and an elongation block (Figure 4E). Therefore, the remaining repression when initiation was prevented (2.6-fold for + TCEIII, +4E1RCat versus 1.1-fold for +TCEIII, -4E1RCat, 2.3-fold repressed) corresponds to a TCE-mediated initiation block (Figure 4E). In summary, TCE-based regulation in wild-type extract comprises 2.3-fold repression of initiation and 3.6-fold repression of elongation (Figure 4F).

In parallel, we assessed the behavior of the dFMRP KD extract. Similarly to the wild-type extract, the addition of TCEIII, but not TCEII, competitor RNA alone almost completely eliminated repression (1.1-fold versus 4.4-fold repression; Figure 4E). In the presence of 4E1RCat, TCEIII, but not TCEII, partially de-repressed *Fluc-3xTCE* RNA (2.4-fold versus 4.4-fold repression, 1.9-fold up-regulated; Figure 4E). Therefore, whereas repression at initiation remained 2.2-fold (2.4-fold for + TCEIII, +4E1RCat versus 1.1-fold for + TCEIII, -4E1RCat, 2.2-fold repressed, not statistically different from the 2.4-fold repression in wild-type extract), repression at elongation was attenuated by 50% (Figure 4F), suggesting that dFMRP specifically represses translation elongation in a Glo/TCE-dependent manner. Consistent with dFMRP acting at the elongation step, *nos* RNA was present in dFMRP immunoprecipitates from polysomal fractions after sucrose density gradient sedimentation (Figure 3D).

### dFMRP stalls ribosomes and decreases elongation rate on *nos*

**Figure 5.**
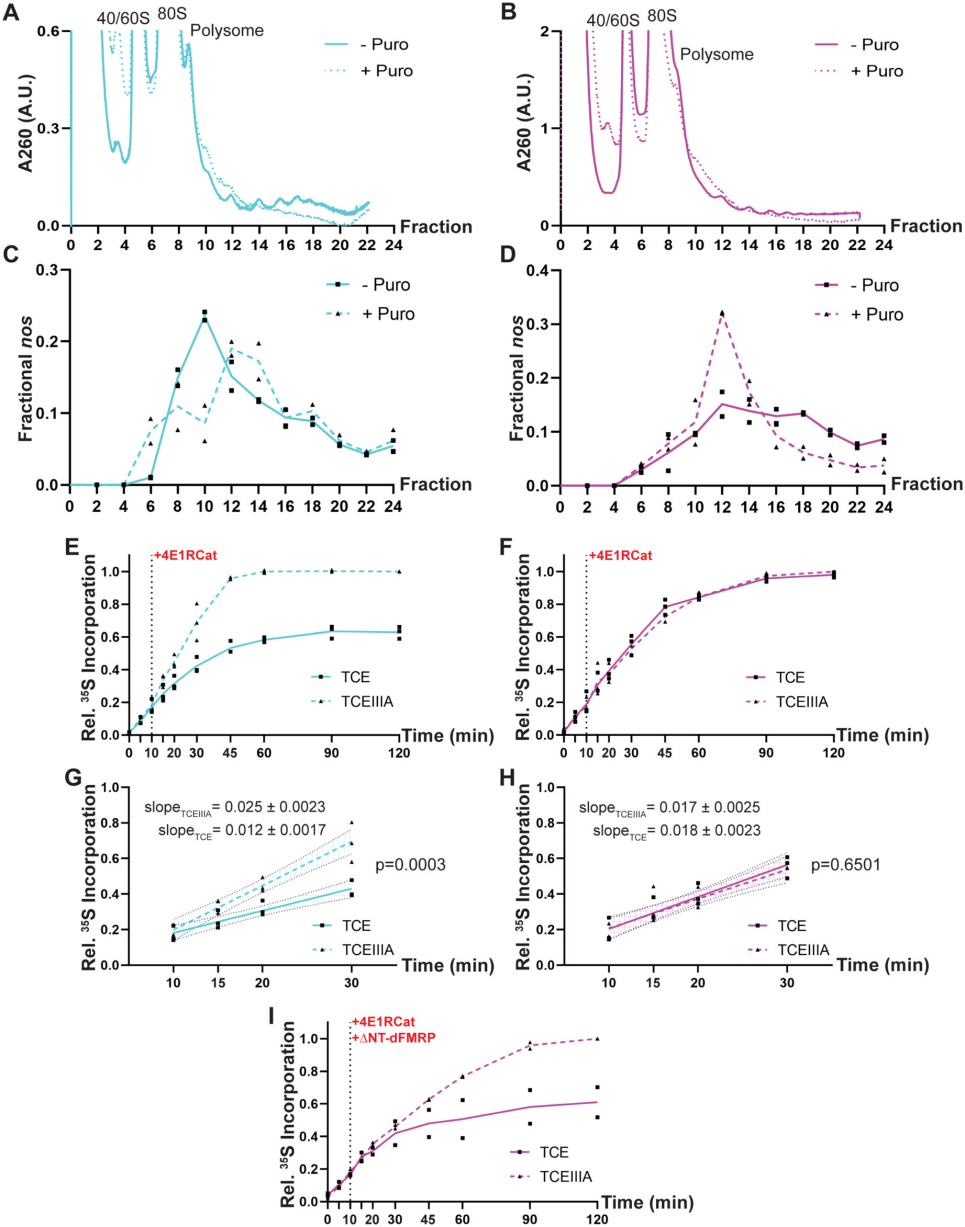
dFMRP stalls ribosomes and decreases elongation rate on *nos*. (**A**, **B**) UV_260_ absorbance profile from representative puromycin release experiment in wild-type (A) and dFMRP KD (B) late-ovary extract after polysome fractionation. The A_260_ range is scaled to better visualize the polysomal fractions. Overall polysome signal decreased when wild-type and dFMRP KD extract were treated by puromycin, which releases elongating but not stalled ribosomes. The same profiles are also used in Supplementary Figure S2A, D. (**C**, **D**) Distribution of *nos* RNA in sucrose gradient fractions of wild-type (C) and dFMRP KD (D) late-ovaries as determined by RT-qPCR. *nos* RNA was partially released from polysomes when treated with puromycin in dFMRP KD but not in wild-type late-ovaries. Relative *nos* level is calculated as 2 to the power of Ct minus Ct of the internal control. The amount of *nos* in each fraction is plotted as a fraction of total *nos* level. Individual data points from two independent puromycin release experiments, each with 3 qPCR technical replicates, are plotted, and their mean values are shown by trend lines. (**E**, **F**) Relative [^35^S]-Met incorporation during translation run-off in wild-type (E) and dFMRP KD (F) *in vitro* translation extract. Translation run-off of *HA-Fluc-3xTCE* RNA resulted in less and slower [^35^S]-Met incorporation than did the translation run-off of *HA-Fluc-3xTCEIIIA* RNA in wild-type, but not in dFMRP KD extract. Run-off began at 10 min (dotted vertical line) with the addition of the initiation inhibitor 4E1RCat (250 μg/ml). Individual data points are plotted and their mean values (n = 3) are shown by trend lines. (**G**, **H**) Linear regression of relative [^35^S]-Met incorporation from 10 to 30 min in wild-type (G) and dFMRP KD (H) extract. The difference in rate of [^35^S]-Met incorporation between the two reporters is significant in wild-type, but not dFMRP KD extract. Regression results are plotted as best-fit line (solid) centered in 95% confidence intervals (shaded area between dotted lines). Slopes are best-fit value ± Std. Error. 95% confidence intervals: 0.0087 to 0.016 (TCE, G); 0.020 to 0.030 (TCEIIIA, G): 0.013 to 0.023 (TCE, H); and 0.011 to 0.022 (TCEIIIA, H). R-squared: 0.85 (TCE, G); 0.92 (TCEIIIA, G); 0.86 (TCE, G) and 0.81 (TCEIIIA, H). (**I**) Relative [^35^S]-Met incorporation during translation run-off in dFMRP KD extract supplemented with purified dFMRP. Purified ΔNT-dFMRP was added to the reaction mixture when translation run-off started (10 min, dotted vertical line). Addition of purified dFMRP decreased both the level and rate of [^35^S]-Met incorporation in the TCE, but not the TCEIIIA, reporter. Relative [^35^S]-Met incorporation is calculated as in (E) and (F). Individual data points are plotted and their mean values (*n* = 2) are shown by trend lines.

The substantial polysome association of *nos* in late-ovaries and the specific inhibition of *nos* translation elongation by dFMRP led us to hypothesize that dFMRP stalls elongating ribosomes on *nos*. To test if dFMRP promotes ribosome stalling on *nos*, we first measured puromycin release of ribosomes on endogenous *nos* in wild-type and dFMRP KD late-ovaries. Puromycin releases actively elongating, but not stalled ribosomes and caused an overall loss of polysomes in both wild-type (Figure 5A) and dFMRP KD (Figure 5B) late-ovaries. The polysome profile of *nos*, however, responded differently to puromycin treatment in the presence or absence of dFMRP. In wild-type late-ovary extract, puromycin treatment did not cause significant loss of polysomal *nos* (Figure 5C, +Puro, Fractions 10 to 24), as compared to the mock-treated extract (Figure 5C, –Puro). In contrast, significant re-distribution of *nos* from heavier to lighter polysomal fractions was observed in puromycin-treated dFMRP KD extract (Figure 5D, +/–Puro). The differential puromycin release of ribosomes from *nos* in wild-type and dFMRP KD extract was not a global effect of dFMRP KD, as ribosomes on a control maternal transcript, *act5c*, were released to a similar extent in both wild-type (Supplementary Figure S3A–C) and dFMRP KD (Supplementary Figure S3D–F) late-ovary extracts. Thus, these results indicate that dFMRP stalls elongating ribosomes on *nos*.

Next, we sought to directly assess if the elongation rate on *nos* is affected by dFMRP in a TCE-dependent manner. To do this, we modified the reporter RNAs to encode an N-terminal triple HA epitope tag (*HA-Fluc-3xTCE* and *HA-Fluc-3xTCEIIIA*), allowing immunoprecipitation of nascent polypeptide chains with anti-HA antibody. We then performed an *in vitro* translation run-off assay with each reporter RNA in the presence the initiation inhibitor 4E1RCat and [^35^S]-Met. Following anti-HA immunoprecipitation, incorporation of [^35^S]-Met into nascent polypeptide chains was monitored by liquid scintillation counting. Because of the low total Met concentration necessary for labeling with [^35^S]-Met (see Materials and Methods), the absolute elongation rate could not be easily estimated. However, we were able to compare qualitatively the elongation rates on the *HA-Fluc-3xTCE* and *HA-Fluc-3xTCEIIIA* RNAs and to determine if the difference in elongation rates depends on dFMRP. Translation run-off with *HA-Fluc-3xTCE* RNA resulted in ∼50% less [^35^S]-Met incorporation than with *HA-Fluc-3xTCEIIIA* in the wild-type late-ovary extract (Figure 5E) but not in the dFMRP KD extract (Figure 5F). This is consistent with the luciferase assay results (Figure 4). We fitted the relative [^35^S]-Met incorporation during first 20 min of the run-off, when neither [^35^S]-Met nor elongating ribosomes were rate-limiting, to a linear model. After regression, the slope for *HA-Fluc-3xTCEIIIA* RNA in the wild-type extract was signifcantly steeper than that for *HA-Fluc-3xTCE*, indicating a faster elongation rate for the mutant reporter RNA (Figure 5G). However, the elongation rates for the two reporters were indistinguishable in the dFMRP KD extract (Figure 5H), consistent with dFMRP impeding elongation. Moreover, adding purified dFMRP, but not BSA, to the dFMRP KD extract slowed [^35^S]-Met incorporation during translation run-off and led to an earlier plateau for *HA-Fluc-3xTCE*, but not for *HA-Fluc-3xTCEIIIA* RNA (Figure 5I, S4). Taken together, results from polysome profiling and [^35^S]-Met incorporation analyses demonstrate that dFMRP stalls elongating ribosomes on *nos* via the TCE.

### qRRM2 is necessary and sufficient for direct recruitment of dFMRP by Glo

**Figure 6.**
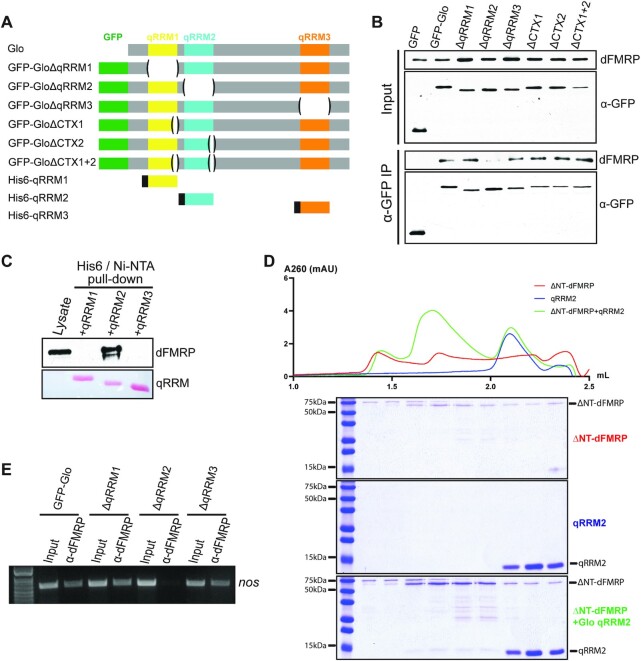
Glo qRRM2 is necessary and sufficient for direct recruitment of dFMRP. (**A**) Schematic representation of Glo protein variants analyzed. Green: GFP tag; yellow: qRRM1; blue: qRRM2; orange: qRRM3; black: His_6_ tag. ΔqRRM1: deletion of residues 47–136; ΔqRRM2: deletion of residues 145–232; ΔqRRM3: deletion of residues 478–559; ΔCTX1: deletion of residues 129–136; ΔCTX2: deletion of residues 226–232; ΔCTX1 + 2: deletion of residues 129–136 and 226–232. His_6_-qRRM1: Glo residues 45–141; His_6_-qRRM2: Glo residues 142–234; His_6_-qRRM3: Glo residues 475–562. (**B**) Anti-dFMRP and anti-GFP immunoblots of input extracts (top two panels) and anti-GFP immunoprecipitates (bottom two panels) from late-ovaries expressing GFP or GFP-Glo variants. dFMRP co-immunprecipitated with all Glo variants except GloΔqRRM2. (**C**) Pull-down assay with His_6_-qRRMs. Top panel, anti-GFP immunoblot to detect GFP-dFMRP (dFMRP) in input extract and after pull-down with qRRM1, qRRM2, or qRRM3. Bottom panel, Ponceau S staining of His_6_-qRRMs in input extract and after pull-down. Only qRRM2 was able to pull down GFP-dFMRP. (**D**) Analytical size-exclusion chromatography binding assays. For chromatograph (top), red profile: 2.5 μM His_6_-ΔNT-dFMRP (ΔNT-dFMRP) alone (the same chromatograph is also used in Supplementary Figure S6A, B); blue profile: 20 μM His_6_-qRRM2 (qRRM2) alone; green profile: 2.5 μM ΔNT-dFMRP + 20 μM qRRM2 (ΔNT-dFMRP + qRRM2). Coomassie stained SDS-PAGE gels below show corresponding fractions from the chromatograph. Mixing ΔNT-dFMRP and qRRM2 resulted in elution of qRRM2 in higher molecular weight fractions. Because Coomassie binding is roughly proportional to the size of the protein (∼53 kDa for ΔNT-dFMRP and ∼10 kDa for qRRM2), the qRRM2 bands in the higher molecular weight fractions represent a significant amount of qRRM2/ΔNT-dFMRP complex. The same ΔNT-dFMRP gel image is also used in Supplementary Figure S6A, B. (**E**) Ethidium bromide-stained gel analysis of RT-PCR for *nos* RNA in anti-dFMRP immunoprecipitates from *glo* null mutant ovaries expressing GFP-Glo and GFP-GloΔqRRM variants. *nos* RNA co-immunoprecipitated with dFMRP when GFP-Glo, GFP-GloΔqRRM1, or GFP-GloΔqRRM3, but not GFP-GloΔqRRM2, was expressed.

Given that Glo lacks known functional domains other than the qRRMs, we hypothesized that Glo interacts with dFMRP through one or more of its qRRMs. To test this, a series of Glo deletion variants were made by removing individual qRRMs. Two of Glo's qRRMs, qRRM1 and qRRM2, have additional alpha helical extensions (CTX) C-terminal to the core RRM fold (18). Since similar CTX structures have been shown to serve as protein-protein interaction platforms in other RRM-containing proteins (41), we also generated variants lacking the CTX residues either individually or in combination (Figure 6A). The deletions were engineered in a functional GFP-Glo transgene, and the resulting transgenes were each integrated at the same chromosomal location.

We then performed anti-GFP IPs from late-ovary extracts to determine whether any of the deletions affected the ability of Glo to interact with dFMRP. Western blot analysis showed that dFMRP co-immunoprecipitated with all Glo variants except GloΔqRRM2 (Figure 6B), indicating that Glo specifically requires qRRM2 to bind to dFMRP. Notably, RNA co-IP analysis showed that all of the deletion variants including qRRM2 bound *nos* (Supplementary Figure S5), confirming that protein structure is maintained and that, consistent with previous biochemical analysis (18), two qRRMs are sufficient for TCE recognition.

Next, we asked if qRRM2 is sufficient for dFMRP interaction. We purified recombinant hexahistidine-tagged qRRMs (18) (Figure 6A) from *E. coli* using Ni-NTA agarose chromatography followed by size-exclusion chromatography. Following purification, qRRMs were immobilized on Ni-NTA agarose and incubated with late-ovary extract expressing GFP-dFMRP. qRRM2, but not qRRM1 or qRRM3, was able to pull down GFP-dFMRP (Figure 6C), indicating that qRRM2 alone is sufficient for Glo-dFMRP interaction.

We then sought to determine if dFMRP binds directly to qRRM2. We expressed and purified a hexahistidine-tagged N-terminally truncated dFMRP protein (ΔNT-dFMRP), which lacks the first 219 amino acid residues containing the nuclear localization signal but is folded and retains full translational repression capability as determined by both cryo-EM and *in vitro* translation (28). Purified ΔNT-dFMRP was incubated with each of the purified qRRM proteins and the mixture was subjected to size-exclusion chromatography. When chromatographed alone, each Glo qRRM behaved as a monomer. However, when mixed with dFMRP, a proportion of the Glo qRRM2 protein eluted in higher molecular weight fractions, together with ΔNT-dFMRP (Figure 6D). Neither Glo qRRM1 nor qRRM3 proteins were shifted to higher molecular weight fractions (Supplementary Figure S6). Together, our results show that interaction of Glo with dFMRP is mediated directly and specifically by qRRM2.

Finally, we tested if Glo is required for recruitment of dFMRP to *nos* by RNA co-IP. dFMRP was immunoprecipitated from *glo* null mutant late-ovaries expressing either GFP-Glo or individual GFP-GloΔqRRM variants, using anti-FMRP antibody. *nos* mRNA was detected by RT-PCR in all dFMRP immunoprecipitates except when the only Glo protein expressed was GFP-GloΔqRRM2 (Figure 6E). The failure of *nos* to co-immunoprecipitate with dFMRP when Glo lacks qRRM2 indicates that qRRM2, and thus the interaction between Glo and dFMRP, is required for dFMRP binding to *nos*.

## DISCUSSION

**Figure 7.**
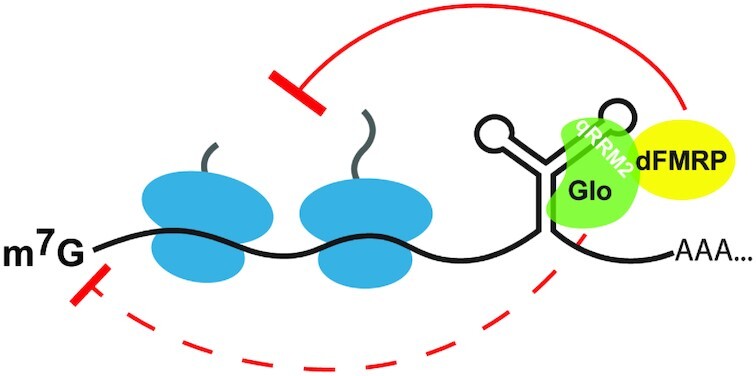
Model of Glo/dFMRP-mediated *nos* regulation. Glo recruits dFMRP to the *nos* TCE via qRRM2 to repress translation elongation of *nos* mRNA.

The *Drosophila* hnRNP F/H homolog, Glo, represses the translation of unlocalized *nos* during late stages of oogenesis to ensure proper anterior-posterior patterning of the embryo. Previous work suggested that Glo imposes both an initiation and a post-initiation block on *nos* translation (20,22), but the molecular mechanism and the nature of the post-initiation block remained unknown. Moreover, how a single protein composed primarily of RNA-binding domains can confer repression by two different mechanisms is unclear. Here, we have begun to fill these gaps by identifying dFMRP as a Glo-interacting protein during late stages of oogenesis and biochemically dissecting its role in *nos* regulation. Our results lead to a new model wherein Glo recruits dFMRP specifically through qRRM2 to repress elongating ribosomes on *nos* (Figure 7) and suggest that Glo's distinct activities are dictated by different effector proteins recruited through different qRRMs.

Since the discovery of FMRP, numerous high-throughput analyses along with transcript-specific studies have uncovered a multiplicity of mechanisms by which it is thought to regulate translation in both neuronal and non-neuronal contexts. These include repression of cap-dependent translation initiation (35,36), modulation of microRNA activity (42,43), and stalling of elongating ribosomes (28,37). Among its large set of targets, FMRP is capable of binding to a wide variety of sequence motifs (44), further complicating the understanding of its target selectivity. In this work, we show that an FMRP-interacting protein recognizing an mRNA signature specific to an individual target mRNA can provide the required target selectivity.

A recent ribosome profiling study found that dFMRP activates translation of a collection of stored mRNAs, particularly those encoding large autism-related proteins, in *Drosophila* oocytes (45). Our work, however, suggests that dFMRP, recruited by specificity factors, can also function as translational repressor of individual targets in oocytes by blocking translation elongation. Indeed, dFMRP has been reported to interact with the 60S ribosomal protein L5, which may prevent tRNA binding during elongation (28). The Glo/TCE-dependent elongation-repressing activity of dFMRP together with the increased ribosome load following dFMRP KD leads to two non-exclusive hypotheses: 1) dFMRP can repress *nos* translation initiation independently of Glo/TCE and/or 2) in the absence of the dFMRP-mediated elongation block, the initiation block, regardless of its dFMRP dependency, is insufficient for complete shutdown of translation. Although we cannot yet distinguish among these possibilities, the latter is consistent with the relative magnitude of the initiation versus elongation-based repression (2.4-fold versus 3.6-fold) measured by our translation run-off assay (Figure 4F).

Whereas Glo-RNA interactions have been characterized in some detail (18), much less is known about Glo-protein interactions and how they contribute to Glo's functional diversity. The lack of apparent functional domains apart from the qRRMs raises the possibility that these RNA-binding domains also mediate protein-protein interactions. Notably, the RRM and its variants have been implicated in RRM-RRM inter-domain packing (46,47) and RRM-protein interactions (48,49). Among these protein-binding RRM family members, the U2AF homology motif (UHM) has emerged as a specialized protein-interacting platform rather than an RNA-interacting platform (50). Interestingly, the mammalian hnRNP F qRRM2 was recently shown to physically interact with FOXP3, which modulates the activity of hnRNP F in regulating alternative splicing (51). Our demonstration that the interaction between Glo and dFMRP also involves qRRM2 suggests that qRRM2 might be an important protein-interaction platform as well as an RNA-binding platform for the hnRNP F/H family more generally. This finding also indicates that despite their structural similarity, the 3 Glo qRRMs differ in their protein binding specificity. Followup studies of other high-confidence Glo-interacting proteins identified by our IP-MS analysis may provide further evidence for differential protein-protein interaction capacity among Glo's qRRMs.

The incorporation of protein-binding and RNA-binding platforms into a single qRRM may enable specific protein interactors to influence RNA target selectivity. Depending on which part of qRRM is used for interaction, a qRRM-interacting protein could affect the accessibility of RNA-binding residues to RNA targets. While the FOXP3-hnRNP F qRRM2 interaction prevents hnRNP F from binding to *BCL-X* pre-mRNA (51), the interaction of SF3b155 to p14, a human RRM-containing U2 and U11/12 snRNP component, exposes secondary RNA-binding residues on the latter (52). Previously, we showed that each Glo qRRM harbors 2 distinct RNA-binding interfaces that allow Glo to recognize two different sequence motifs: a single-stranded G-tract and a double-stranded UA-rich motif (18). The integration of both binding modes into a single qRRM and the combination of 3 qRRMs are thought to diversify the RNA target repertoire of Glo. Both the G-tract and UA-rich motif binding modes are required for Glo-TCE interaction, and hence *nos* regulation, as well as for regulation of as yet unknown targets required for viability. By contrast, only the G-tract binding mode is required for Glo's function as a putative splicing regulator in dorsal-ventral patterning and ovarian nurse cell chromatin structure remodeling. It remains to be determined if and how binding of dFMRP affects the RNA-binding affinity and specificity of qRRM2. Regardless, the choice of protein interactor could influence the combinatorial use of the 6 RNA-binding interfaces of Glo. For example, binding of Glo to dFMRP or other *nos* regulatory factors may leave both RNA-binding surfaces exposed, favoring *nos* TCE recognition and translational repression, whereas binding of Glo to Hrp48 and Hfp may leave only the G-tract binding residues exposed, favoring a different subset of targets for alternative splicing.

Using *nos* regulation as a model, we have shown that Glo represses translation elongation by interacting with a polysome-associated protein, dFMRP, and that Glo's RNA-binding qRRM domains also mediate protein-protein interaction. Since Glo is a multi-functional post-transcriptional regulator, the same principles could also be applied to its other activities where integration of multiple regulatory modes into a single node is required. Identification of such targets and additional regulatory factors involved will forward our understanding of how qRRMs of Glo contribute to its functional diversity and, more generally, how RNA-binding proteins confer post-transcriptional gene regulation.

## MATERIALS AND CORRESPONDENCE

Reagent requests and other correspondence should be addressed to Elizabeth R. Gavis.

## DATA AVAILABILITY

The datasets generated or analyzed during this study are included in this published article (and its supplementary information files). Data are available via ProteomeXchange with identifier PXD033174 and PXD033175.

## Supplementary Material

gkac500_Supplemental_Files

## References

[1] Richter J.D. , Lasko P. Translational control in oocyte development. Cold Spring Harb. Perspect. Biol. 2011; 3:a002758. 21690213 10.1101/cshperspect.a002758 PMC3181033

[2] Tadros W. , Lipshitz H.D. Setting the stage for development: mRNA translation and stability during oocyte maturation and egg activation in *Drosophil**a*. Dev. Dyn. 2005; 232:593–608. 15704150 10.1002/dvdy.20297

[3] Evans T.C. , Hunter C.P. Translational control of maternal RNAs. WormBook. 2005; 1–11. 10.1895/wormbook.1.34.1 PMC4780918 18050410

[4] St Johnston D. Moving messages: the intracellular localization of mRNAs. Nat. Rev. Mol. Cell Biol. 2005; 6:363–375. 15852043 10.1038/nrm1643

[5] Berleth T. , Burri M., Thoma G., Bopp D., Richstein S., Frigerio G., Noll M., Nüsslein-Volhard C. The role of localization of *bicoid* RNA in organizing the anterior pattern of the *Drosophila* embryo. EMBO J. 1988; 7:1749–1756. 2901954 10.1002/j.1460-2075.1988.tb03004.x PMC457163

[6] Bergsten S.E. , Gavis E.R. Role for mRNA localization in translational activation but not spatial restriction of *nanos* RNA. Development. 1999; 126:659–669. 9895314 10.1242/dev.126.4.659

[7] Driever W. , Nüsslein-Volhard C. A gradient of *bicoid* protein in *Drosophila* embryos. Cell. 1988; 54:83–93. 3383244 10.1016/0092-8674(88)90182-1

[8] Forrest K.M. , Gavis E.R. Live imaging of endogenous RNA reveals a diffusion and entrapment mechanism for *nanos* mRNA localization in *Drosophil**a*. Curr. Biol. 2003; 13:1159–1168. 12867026 10.1016/s0960-9822(03)00451-2

[9] Forrest K.M. , Clark I.E., Jain R.A., Gavis E.R. Temporal complexity within a translational control element in the *nanos* mRNA. Development. 2004; 131:5849–5857. 15525666 10.1242/dev.01460

[10] Gavis E.R. , Lehmann R. Localization of *nanos* RNA controls embryonic polarity. Cell. 1992; 71:301–313. 1423595 10.1016/0092-8674(92)90358-j

[11] Wang C. , Lehmann R. Nanos is the localized posterior determinant in *Drosophil**a*. Cell. 1991; 66:637–647. 1908748 10.1016/0092-8674(91)90110-k

[12] Wang C. , Dickinson L.K., Lehmann R. Genetics of *nanos* localization in *Drosophil**a*. Dev. Dyn. 1994; 199:103–115. 7515724 10.1002/aja.1001990204

[13] Dahanukar A. , Wharton R.P. The Nanos gradient in *Drosophila* embryos is generated by translational regulation. Genes Dev. 1996; 10:2610–2620. 8895662 10.1101/gad.10.20.2610

[14] Gavis E.R. , Lunsford L., Bergsten S.E., Lehmann R. A conserved 90 nucleotide element mediates translational repression of *nanos* RNA. Development. 1996; 122:2791–2800. 8787753 10.1242/dev.122.9.2791

[15] Smibert C.A. , Wilson J.E., Kerr K., Macdonald P.M. Smaug protein represses translation of unlocalized *nanos* mRNA in the *Drosophila* embryo. Genes Dev. 1996; 10:2600–2609. 8895661 10.1101/gad.10.20.2600

[16] Crucs S. , Chatterjee S., Gavis E.R. Overlapping but distinct RNA elements control repression and activation of *nanos* translation. Mol. Cell. 2000; 5:457–467. 10882131 10.1016/s1097-2765(00)80440-2

[17] Kalifa Y. , Huang T., Rosen L.N., Chatterjee S., Gavis E.R. Glorund, a *Drosophila* hnRNP F/H homolog, is an ovarian repressor of *nanos* translation. Dev. Cell. 2006; 10:291–301. 16516833 10.1016/j.devcel.2006.01.001

[18] Tamayo J.V. , Teramoto T., Chatterjee S., Hall T.M.T., Gavis E.R. The *Drosophila* hnRNP F/H homolog Glorund uses two distinct RNA-binding modes to diversify target recognition. Cell Rep. 2017; 19:150–161. 28380354 10.1016/j.celrep.2017.03.022 PMC5392723

[19] Dahanukar A. , Walker J.A., Wharton R.P. Smaug, a novel RNA-binding protein that operates a translational switch in *Drosophil**a*. Mol. Cell. 1999; 4:209–218. 10488336 10.1016/s1097-2765(00)80368-8

[20] Andrews S. , Snowflack D.R., Clark I.E., Gavis E.R. Multiple mechanisms collaborate to repress *nanos* translation in the *Drosophila* ovary and embryo. RNA. 2011; 17:967–977. 21460235 10.1261/rna.2478611 PMC3078745

[21] Nelson M.R. , Leidal A.M., Smibert C.A. *Drosophila* Cup is an eIF4E-binding protein that functions in Smaug-mediated translational repression. EMBO J. 2004; 23:150–159. 14685270 10.1038/sj.emboj.7600026 PMC1271664

[22] Clark I.E. , Wyckoff D., Gavis E.R. Synthesis of the posterior determinant Nanos is spatially restricted by a novel cotranslational regulatory mechanism. Curr. Biol. 2000; 10:1311–1314. 11069116 10.1016/s0960-9822(00)00754-5

[23] Gehrke S. , Wu Z., Klinkenberg M., Sun Y., Auburger G., Guo S., Lu B. PINK1 and Parkin control localized translation of respiratory chain component mRNAs on mitochondria outer membrane. Cell Metab. 2015; 21:95–108. 25565208 10.1016/j.cmet.2014.12.007 PMC4455944

[24] Kalifa Y. , Armenti S.T., Gavis E.R. Glorund interactions in the regulation of *gurken* and *oskar* mRNAs. Dev. Biol. 2009; 326:68–74. 19013444 10.1016/j.ydbio.2008.10.032 PMC2839899

[25] Kolasa A.M. , Bhogal J.K., DiAngelo J.R. The heterogeneous nuclear ribonucleoprotein (hnRNP) Glorund functions in the *Drosophila* fat body to regulate lipid storage and transport. Biochem. Biophys. Rep. 2021; 25:100919. 33537463 10.1016/j.bbrep.2021.100919 PMC7838711

[26] Chou T.-B. , Noll E., Perrimon N. Autosomal *P[ovoD1]* dominant female-sterile insertions in *Drosophila* and their use in generating germ-line chimeras. Development. 1993; 119:1359–1369. 8306893 10.1242/dev.119.4.1359

[27] Sudhakaran I.P. , Hillebrand J., Dervan A., Das S., Holohan E.E., Hulsmeier J., Sarov M., Parker R., VijayRaghavan K., Ramaswami M. FMRP and Ataxin-2 function together in long-term olfactory habituation and neuronal translational control. Proc. Natl. Acad. Sci. U.S.A. 2014; 111:E99–E108. 24344294 10.1073/pnas.1309543111 PMC3890871

[28] Chen E. , Sharma M.R., Shi X., Agrawal R.K., Joseph S. Fragile x mental retardation protein regulates translation by binding directly to the ribosome. Mol. Cell. 2014; 54:407–417. 24746697 10.1016/j.molcel.2014.03.023 PMC4019695

[29] Wan L. , Dockendorff T.C., Jongens T.A., Dreyfuss G. Characterization of dFMR1, a *Drosophila**melanogaster* homolog of the fragile x mental retardation protein. Mol. Cell. Biol. 2000; 20:8536–8547. 11046149 10.1128/mcb.20.22.8536-8547.2000 PMC102159

[30] Zhang Y.Q. , Bailey A.M., Matthies H.J.G., Renden R.B., Smith M.A., Speese S.D., Rubin G.M., Broadie K. *Drosophila* fragile X-related gene regulates the MAP1B homolog Futsch to control synaptic structure and function. Cell. 2001; 107:591–603. 11733059 10.1016/s0092-8674(01)00589-x

[31] Papoulas O. , Monzo K.F., Cantin G.T., Ruse C., Yates J.R. 3rd, Ryu Y.H., Sisson J.C. dFMRP and Caprin, translational regulators of synaptic plasticity, control the cell cycle at the *Drosophila* mid-blastula transition. Development. 2010; 137:4201–4209. 21068064 10.1242/dev.055046 PMC2990211

[32] Kuniyoshi H. , Baba K., Ueda R., Kondo S., Awano W., Juni N., Yamamoto D *lingerer*, a *Drosophila* gene involved in initiation and termination of copulation, encodes a set of novel cytoplasmic proteins. Genetics. 2002; 162:1775–1789. 12524348 10.1093/genetics/162.4.1775 PMC1462391

[33] Kimura S. , Sakakibara Y., Sato K., Ote M., Ito H., Koganezawa M., Yamamoto D The *Drosophila* lingerer protein cooperates with Orb2 in long-term memory formation. J. Neurogenet. 2015; 29:8–17. 24913805 10.3109/01677063.2014.917644

[34] Bansal P. , Madlung J., Schaaf K., Macek B., Bono F. An interaction network of RNA-binding proteins involved in *Drosophila* oogenesis. Mol. Cell. Proteomics. 2020; 19:1485–1502. 32554711 10.1074/mcp.RA119.001912 PMC8143644

[35] Schenck A. , Bardoni B., Moro A., Bagni C., Mandel J. A highly conserved protein family interacting with the fragile x mental retardation protein (FMRP) and displaying selective interactions with FMRP-related proteins FXR1P and FXR2P. Proc. Natl. Acad. Sci. U.S.A. 2001; 98:8844–8849. 11438699 10.1073/pnas.151231598 PMC37523

[36] Napoli I. , Mercaldo V., Boyl P.P., Eleuteri B., Zalfa F., De Rubeis S., Di Marino D., Mohr E., Massimi M., Falconi M. et al. The fragile x syndrome protein represses activity-dependent translation through CYFIP1, a new 4E-BP. Cell. 2008; 134:1042–1054. 18805096 10.1016/j.cell.2008.07.031

[37] Darnell J.C. , Van Driesche S.J., Zhang C., Hung K.Y., Mele A., Fraser C.E., Stone E.F., Chen C., Fak J.J., Chi S.W. et al. FMRP stalls ribosomal translocation on mRNAs linked to synaptic function and autism. Cell. 2011; 146:247–261. 21784246 10.1016/j.cell.2011.06.013 PMC3232425

[38] Kronja I. , Yuan B., Eichhorn S.W., Dzeyk K., Krijgsveld J., Bartel D.P., Orr-Weaver T.L. Widespread changes in the posttranscriptional landscape at the *Drosophila* oocyte-to-embryo transition. Cell Rep. 2014; 7:1495–1508. 24882012 10.1016/j.celrep.2014.05.002 PMC4143395

[39] Jeske M. , Moritz B., Anders A., Wahle E. Smaug assembles an ATP-dependent stable complex repressing *nanos* mRNA translation at multiple levels. EMBO J. 2011; 30:90–103. 21081899 10.1038/emboj.2010.283 PMC3020108

[40] Cencic R. , Hall D.R., Robert F., Du Y., Min J., Li L., Qui M., Lewis I., Kurtkaya S., Dingledine R. et al. Reversing chemoresistance by small molecule inhibition of the translation initiation complex eIF4F. Proc. Natl. Acad. Sci. U.S.A. 2011; 108:1046–1051. 21191102 10.1073/pnas.1011477108 PMC3024666

[41] Trowitzsch S. , Weber G., Luhrmann R., Wahl M.C. An unusual RNA recognition motif acts as a scaffold for multiple proteins in the pre-mRNA retention and splicing complex. J. Biol. Chem. 2008; 283:32317–32327. 18809678 10.1074/jbc.M804977200

[42] Jin P. , Zarnescu D.C., Ceman S., Nakamoto M., Mowrey J., Jongens T.A., Nelson D.L., Moses K., Warren S.T. Biochemical and genetic interaction between the fragile x mental retardation protein and the microRNA pathway. Nat. Neurosci. 2004; 7:113–117. 14703574 10.1038/nn1174

[43] Muddashetty R.S. , Nalavadi V.C., Gross C., Yao X., Xing L., Laur O., Warren S.T., Bassell G.J. Reversible inhibition of PSD-95 mRNA translation by miR-125a, FMRP phosphorylation, and mGluR signaling. Mol. Cell. 2011; 42:673–688. 21658607 10.1016/j.molcel.2011.05.006 PMC3115785

[44] Anderson B.R. , Chopra P., Suhl J.A., Warren S.T., Bassell G.J. Identification of consensus binding sites clarifies FMRP binding determinants. Nucl. Acids Res. 2016; 44:6649–6659. 27378784 10.1093/nar/gkw593 PMC5001617

[45] Greenblatt E.J. , Spradling A.C. Fragile x mental retardation 1 gene enhances the translation of large autism-related proteins. Science. 2018; 361:709–712. 30115809 10.1126/science.aas9963 PMC6905618

[46] Handa N. , Nureki O., Kurimoto K., Kim I., Sakamoto H., Shimura Y., Muto Y., Yokoyama S. Structural basis for recognition of the *tra* mRNA precursor by the Sex-lethal protein. Nature. 1999; 398:579–585. 10217141 10.1038/19242

[47] Wang X. , Hall T.M.T. Structural basis for recognition of AU-rich element RNA by the HuD protein. Nat. Struct. Biol. 2001; 8:141–145. 11175903 10.1038/84131

[48] Rideau A.P. , Gooding C., Simpson P.J., Monie T.P., Lorenz M., Huttelmaier S., Singer R.H., Matthews S., Curry S., Smith C.W. A peptide motif in Raver1 mediates splicing repression by interaction with the PTB RRM2 domain. Nat. Struct. Mol. Biol. 2006; 13:839–848. 16936729 10.1038/nsmb1137

[49] ElAntak L. , Tzakos A.G., Locker N., Lukavsky P.J. Structure of eIF3b RNA recognition motif and its interaction with eIF3j: structural insights into the recruitment of eIF3b to the 40 S ribosomal subunit. J. Biol. Chem. 2007; 282:8165–8174. 17190833 10.1074/jbc.M610860200

[50] Loerch S. , Kielkopf C.L. Unmasking the U2AF homology motif family: a bona fide protein-protein interaction motif in disguise. RNA. 2016; 22:1795–1807. 27852923 10.1261/rna.057950.116 PMC5113200

[51] Du J. , Wang Q., Ziegler S.F., Zhou B. FOXP3 interacts with hnRNPF to modulate pre-mRNA alternative splicing. J. Biol. Chem. 2018; 293:10235–10244. 29773655 10.1074/jbc.RA117.001349 PMC6028976

[52] Kuwasako K. , Dohmae N., Inoue M., Shirouzu M., Taguchi S., Guntert P., Seraphin B., Muto Y., Yokoyama S. Complex assembly mechanism and an RNA-binding mode of the human p14-SF3b155 spliceosomal protein complex identified by NMR solution structure and functional analyses. Proteins. 2008; 71:1617–1636. 18076038 10.1002/prot.21839

