# Supplementary material for: The Drosophila hnRNP F/H homolog Glorund recruits dFMRP to inhibit nanos translation elongation
Source: Nucleic Acids Res. 2022 Jun 14;50(12):7067–83. doi: 10.1093/nar/gkac500 (PMC9262583; doi:10.1093/nar/gkac500)
Supplement: gkac500_Supplemental_Files [file gkac500_supplemental_files.zip › Supplemental Figures.pdf]

Table S1. Summary of IP-MS results. The “Analysis” tab summarizes the results from the native and crosslinked IP-MS experiments. Column B, name of identified proteins. Column C, accession number. Column D, molecular weight in kDa. Column E and F, total spectra count in control (MCP-GFP) versus Glo (GFP-Glo) IP-MS under native conditions. Columns G and H, total spectra count in un-crosslinked control (unXlink) versus experimental, crosslinked (Xlink) IP-MS. Columns I and J, fold enrichment (experimental over control group) in Native IP-MS and Crosslinked IP-MS. Columns K and L, IP-MS interactor is considered enriched (YES) if > 2-fold enriched in Native or Crosslinked IP-MS datasets. Column M, IP-MS interactor is considered high-confidence (YES) if enriched in both IP-MS datasets. Column N, interactors also identified by Bansal et al. [34]: YES if high-confidence interactor is also > 2-fold enriched in the Bansal dataset; (YES) if > 2-fold enriched interactor from native IP-MS is also > 2-fold enriched in the Bansal dataset. Columns O-Q, number of proteins enriched in either or both IP-MS experiments (O and P) and number of overlapping interactors in the Bansal dataset. The “Raw Scaffold Output” sheet is directly exported from the Scaffold file combining results from both IP-MS experiments.

Figure S1

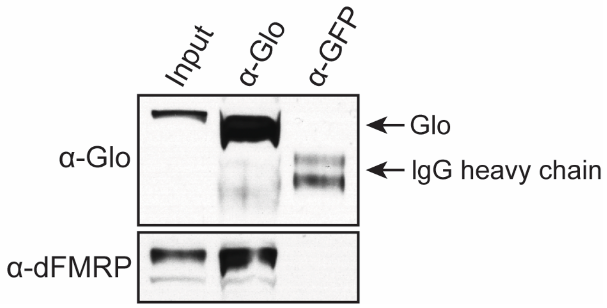

Figure S1. Immunoprecipitation of endogenous Glo. Immunoblots of extract prior to anti-Glo immunoprecipitation (input), anti-Glo immunoprecipitates (α-Glo), and anti-GFP immunoprecipitates (α-GFP) from wild-type *Drosophila* late-ovaries. Immunoblots were probed with anti-Glo antibody (top panel) or anti-dFMRP antibody (bottom panel). Endogenous dFMRP co-immunoprecipitated with endogenous Glo.

Figure S2

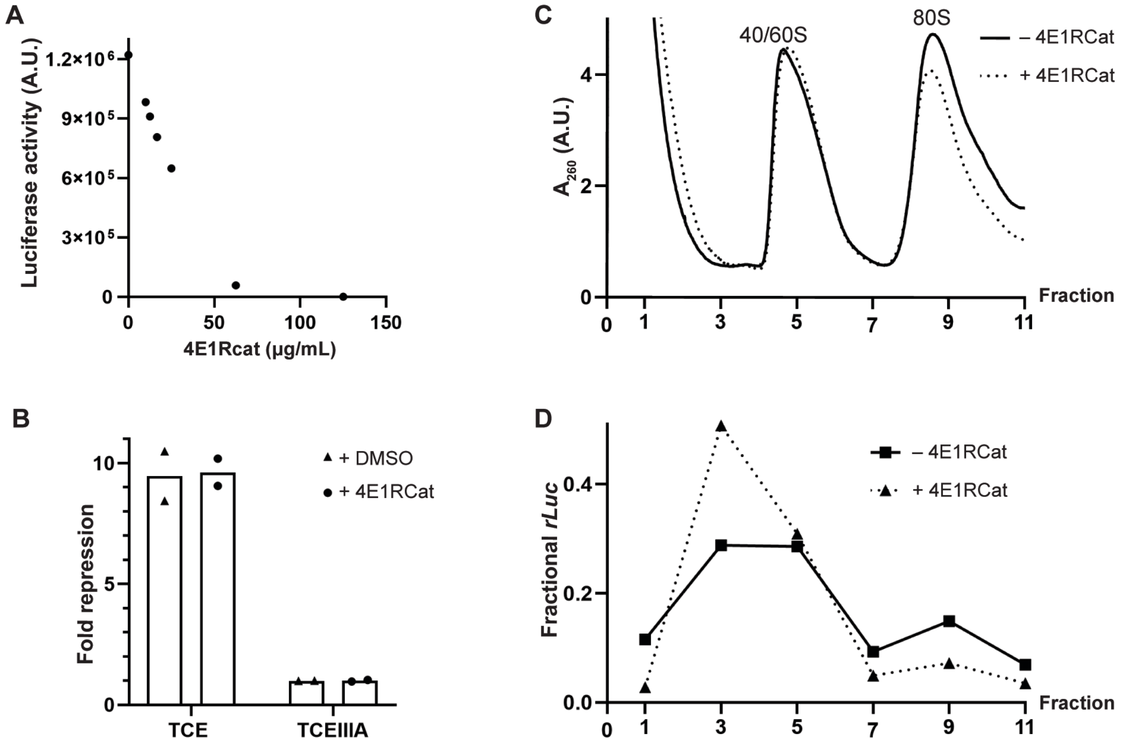

Figure S2. 4E1RCat inhibits translation of reporter RNA and does not affect TCE-mediated repression. **(A)** *Fluc-tub3'UTR* was incubated in rabbit reticulocyte lysate in the presence of increasing concentrations of 4E1RCat for 2 hours at 28°C. 4E1RCat at 50 µg/mL concentration was able to reduce reporter activity by ~90%. (**B)** Standard *in vitro* translation reaction with *Fluc-3xTCE* (TCE) and *Fluc-3xTCEIIIA* (TCEIIIA) reporter RNAs in the absence (+DMSO) or presence (+4E1RCat) of 50 µg/mL 4E1RCat. TCE-mediated repression was not affected by addition of 4E1RCat. (**C)** UV_{260} absorbance profile of wild-type late-ovary translation extract after polysome fractionation. 50 µg/mL 4E1RCat (final concentration) was added to the translation extract in the presence of cycloheximide. The 80S peak in the profile is due to pre-existing 80S ribosomes in the extract. (**D)** Distribution of *Renilla* luciferase RNA (normalized to internal control) in the fractions shown in (C). 50 µg/mL 4E1RCat effectively prevented 80S formation on *rluc* RNA after 30 min incubation.

Figure S3

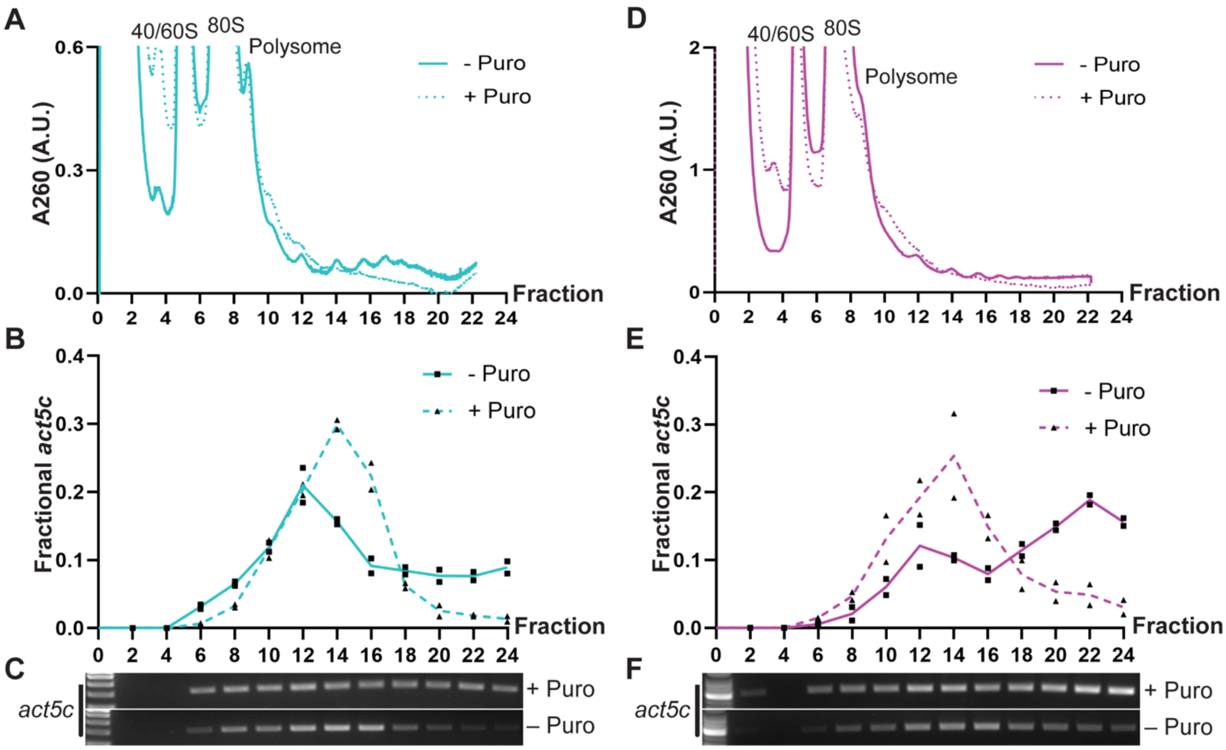

Figure S3. Puromycin releases *act5c* RNA from polysomes in both wild-type and dFMRP KD late-ovaries. **(A-B)** Puromycin release experiment with wild-type late-ovaries. Representative UV_{260} absorbance profile after polysome fractionation (A) is also used in Fig. 5A. The A_{260} range is scaled to better visualize the polysomal fractions. Distribution of *act5c* RNA is quantified by RT-qPCR (B) and is also visualized by ethidium bromide-stained agarose gel electrophoresis of RT-PCR products (C). **(D-F)** Puromycin release experiment with dFMRP KD late-ovaries. Representative UV_{260} absorbance profile after polysome fractionation (D) is also used in Fig. 5B. Distribution of *act5c* RNA is quantified by RT-qPCR (E) and is also visualized by ethidium bromide-stained agarose gel electrophoresis of RT-PCR product (F). Individual data points (B, E) are plotted and their mean values (n = 2) are shown by trend lines. *act5c* RNA was released from polysomes by puromycin in both wild-type and dFMRP KD late-ovaries.

Figure S4

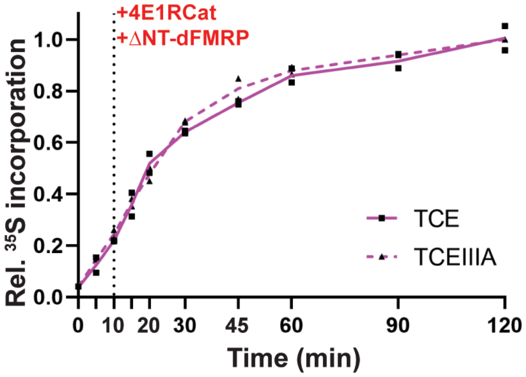

Figure S4. BSA does not affect translation run-off on *HA-Fluc-3xTCE* or *HA-Fluc-3xTCEIIIA* RNA in dFMRP KD late-ovary translation extract. Relative [^{35}S]-Met incorporation during translation run-off in dFMRP KD extract with BSA was added to the reaction mixture (final concentration = 1 µM) at the start of translation run-off (10 min, dotted vertical line). Addition of BSA did not affect the level or rate of [^{35}S]-Met incorporation for either reporter. Relative [^{35}S]-Met incorporation is calculated as in Fig. 5E, F. Individual data points 2 independent labelling experiments, with mean values shown by trend lines.

Figure S5

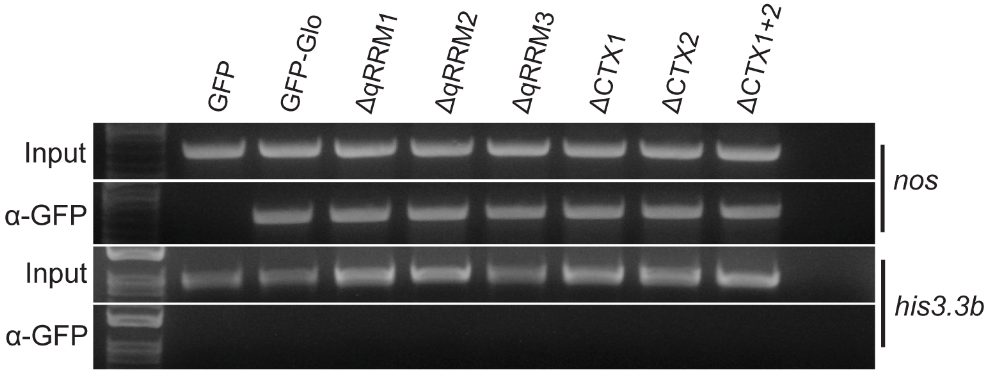

Figure S5. Deletion of individual qRRM or CTX does not affect *nos* binding. RT-PCR for *nos* and control *his3.3b* RNA in anti-GFP immunoprecipitates from late-ovaries expressing GFP-tagged Glo variants or GFP alone in a wild-type background. PCR products were analyzed on an ethidium stained agarose gel. *nos*, but not *his3.3b* RNA co-immunoprecipitated with Glo and all variants.

Figure S6

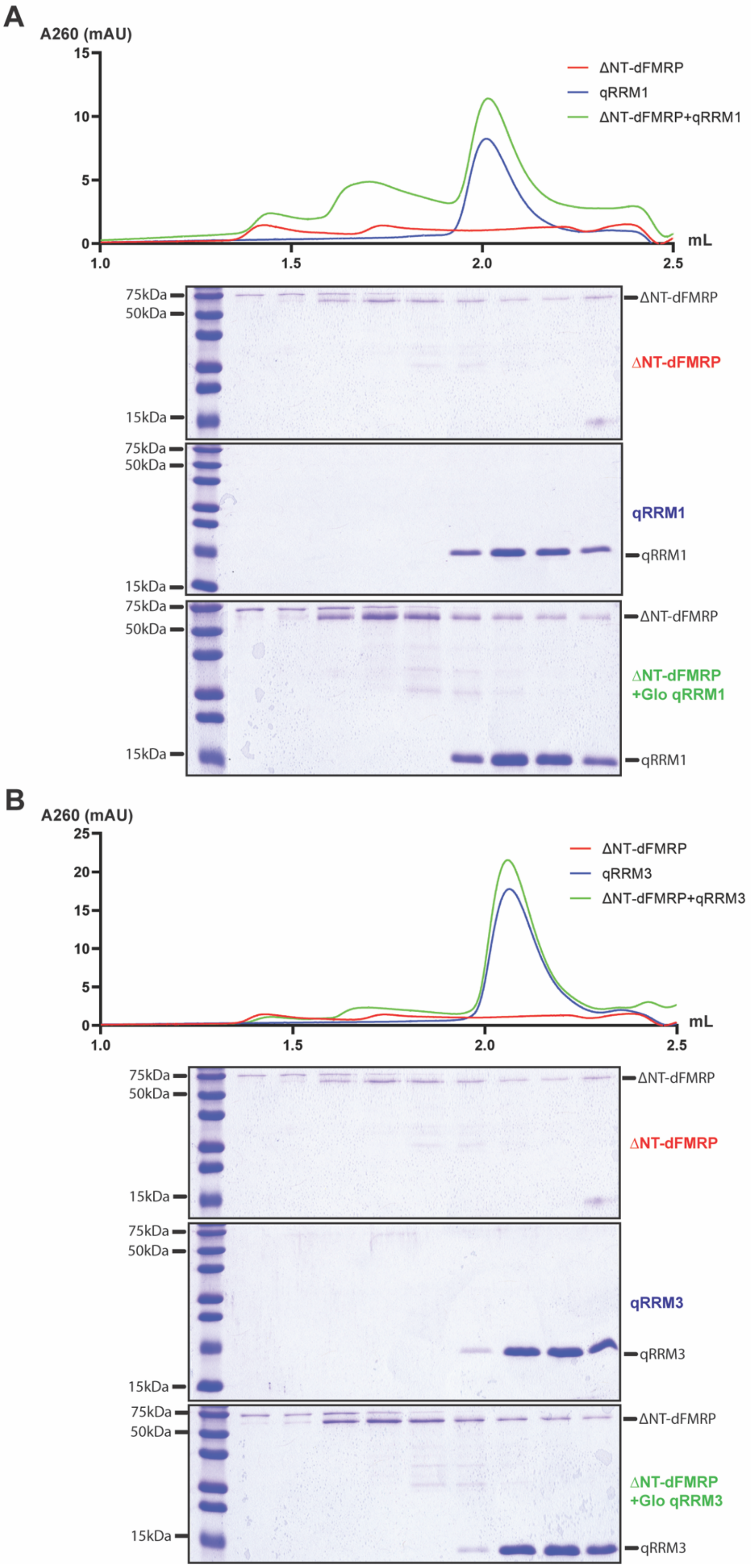

A
A260 (mAU)
15
10
5
0
1.0
1.5
2.0
2.5
mL
ΔNT-dFMRP
qRRM1
ΔNT-dFMRP+qRRM1
75kDa
50kDa
15kDa
ΔNT-dFMRP
qRRM1
ΔNT-dFMRP
+Glo qRRM1
B
A260 (mAU)
25
20
15
10
5
0
1.0
1.5
2.0
2.5
mL
ΔNT-dFMRP
qRRM3
ΔNT-dFMRP+qRRM3
75kDa
50kDa
15kDa
ΔNT-dFMRP
qRRM3
ΔNT-dFMRP
+Glo qRRM3

Figure S6. Glo qRRM1 and qRRM3 do not bind to dFMRP. **(A)** Chromatograph of analytical size-exclusion chromatography binding assay (top), red profile: 2.5 µM His_6-ΔNT-dFMRP (ΔNT-dFMRP) alone (the same profile is also used in Fig. 6D and Fig. S5A); blue profile: 20 µM His_6-qRRM2 (qRRM2) alone; green profile: 2.5 µM ΔNT-dFMRP + 20 µM qRRM2 (ΔNT-dFMRP+qRRM2). Coomassie stained SDS-PAGE gels below show corresponding fractions from chromatograph. Mixing ΔNT-dFMRP and qRRM2 resulted in elution of qRRM2 in higher molecular weight fractions. The same ΔNT-dFMRP gel image is also used in Fig. 6D and Fig. S5B. **(B)** Chromatograph of analytical size-exclusion chromatography binding assay (top), red profile: 2.5 µM His_6-ΔNT-dFMRP (ΔNT-dFMRP) alone (the same profile is also used in Fig. 6D and Fig. S5A); blue profile: 20 µM His_6-qRRM3 (qRRM3) alone; green profile: 2.5 µM ΔNT-dFMRP + 20 µM qRRM3 (ΔNT-dFMRP+qRRM3). Coomassie stained SDS-PAGE gels below showing fractions from (B). The same ΔNT-dFMRP gel image is also used in Fig. 6D and Fig. S5A.
